# Supplementary figures and images for: Clinical features and prognostic factors of IV combined small cell lung cancer: A propensity score matching analysis
Source: PLoS One. 2024 Nov 8;19(11):e0313221. doi: 10.1371/journal.pone.0313221 (PMC11548789; doi:10.1371/journal.pone.0313221)

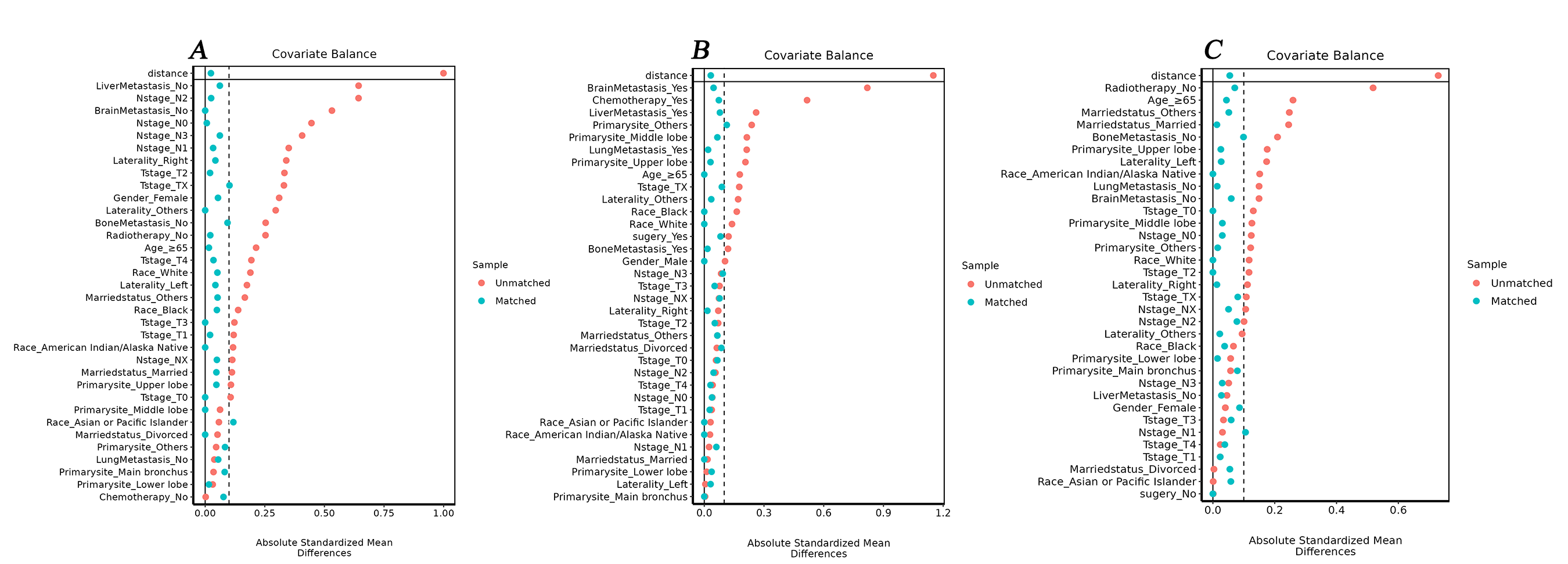

Supplement: S1 Fig — A: 1:4 match of surgery and non-surgical treatment groups; B: 1:1 match of radiotherapy and non-radiotherapy treatment groups; C: 1:1 match of chemotherapy and non-chemotherapy treatment groups. (TIF) [file pone.0313221.s001.tif]

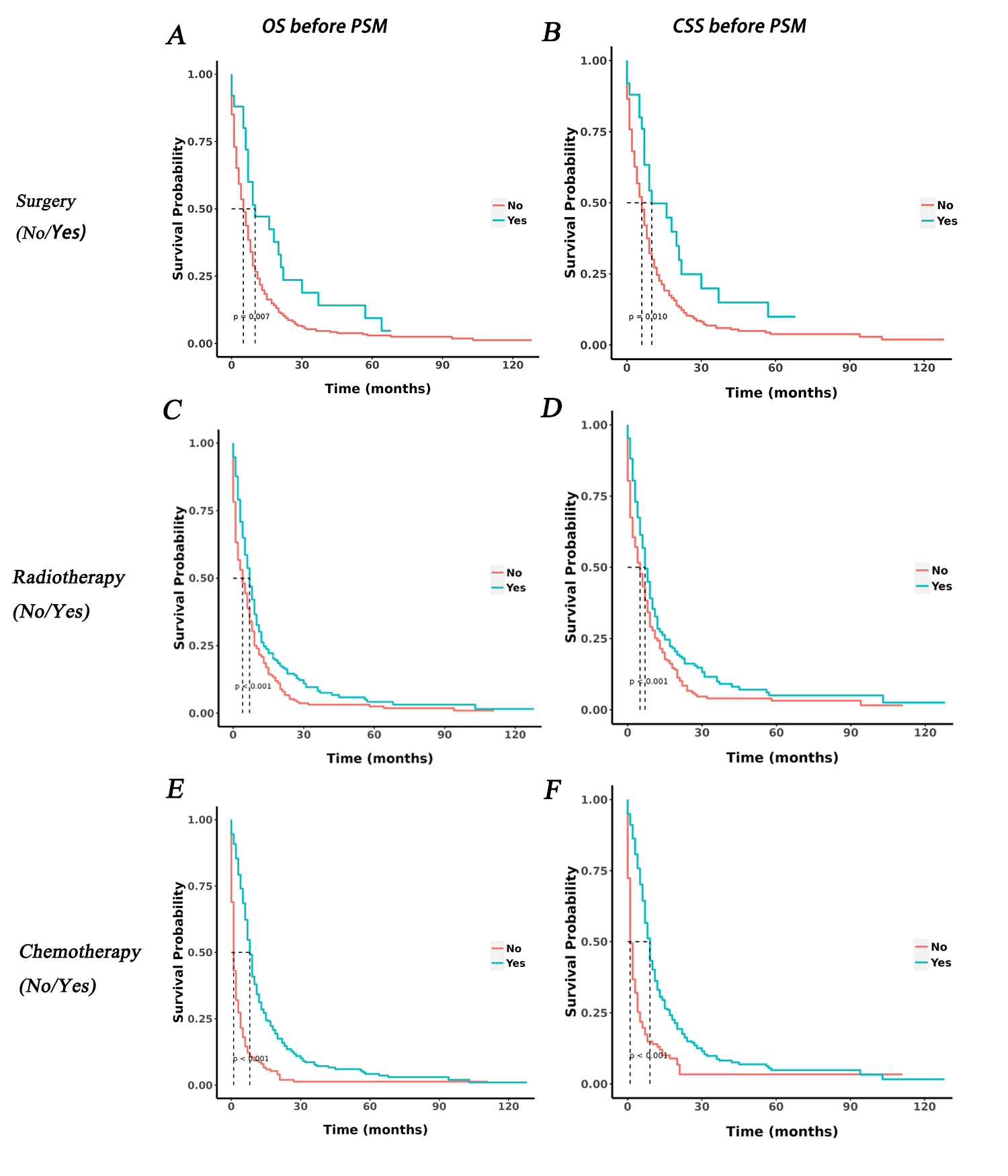

Supplement: S2 Fig — A, B: KM curve of OS and CSS in surgery and non-surgical treatment groups. C, D: KM curve of OS and CSS in radiotherapy and non-radiotherapy treatment groups. E, F: KM curve of OS and CSS in chemotherapy and non-chemotherapy treatment groups. (TIF) [file pone.0313221.s002.tif]

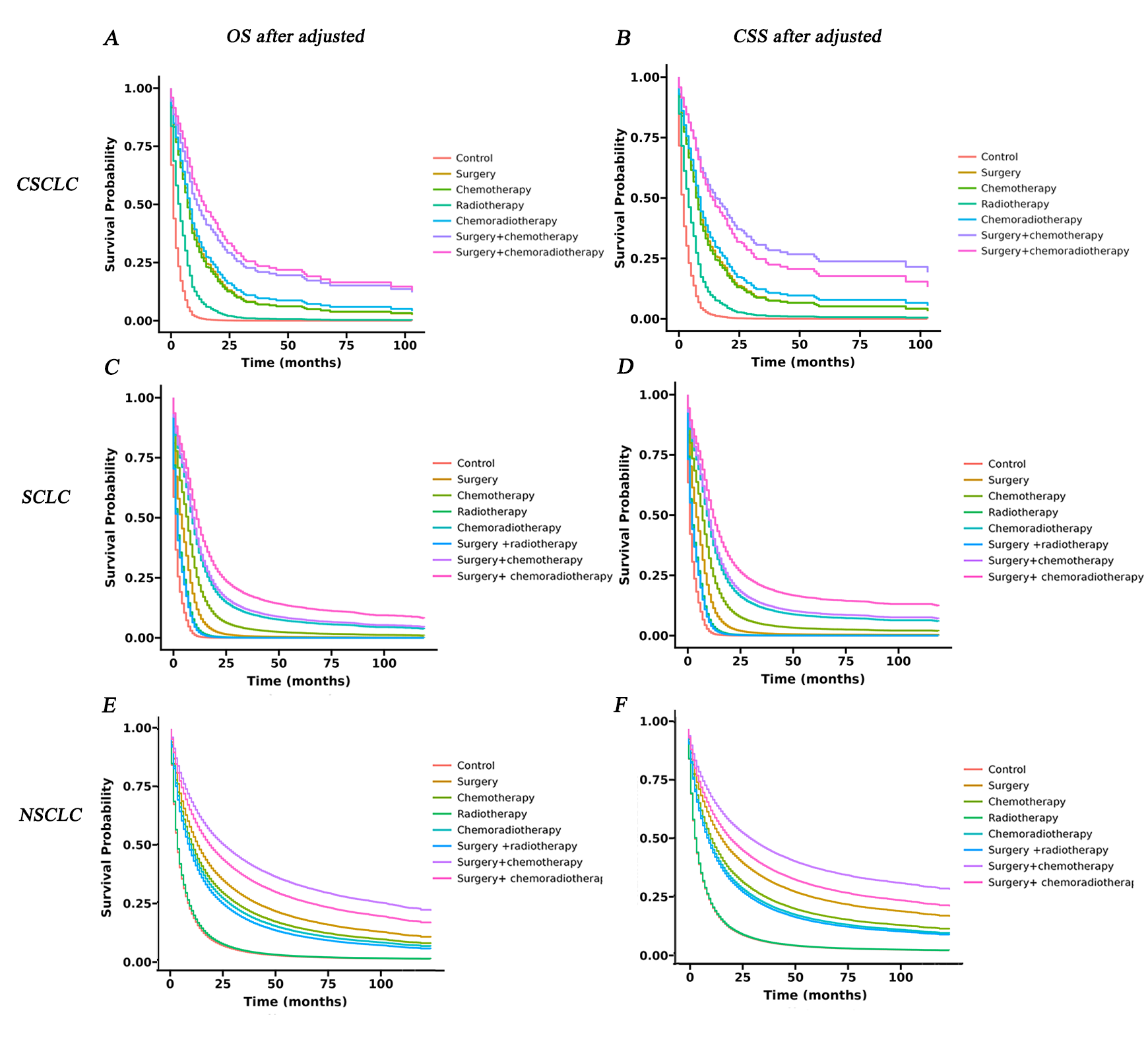

Supplement: S3 Fig — A, B KM curves of OS and CSS for IV CSCLC. C, D: KM curves of OS and CSS for IV SCLC. E, F: KM curves of OS and CSS for IV NSCLC. (TIF) [file pone.0313221.s003.tif]
