# Supplementary material for: Clinical features and prognostic factors of IV combined small cell lung cancer: A propensity score matching analysis
Source: PLoS One. 2024 Nov 8;19(11):e0313221. doi: 10.1371/journal.pone.0313221 (PMC11548789; doi:10.1371/journal.pone.0313221)
Supplement: S1 Table — (DOCX) [file pone.0313221.s004.docx]

S1Table: Univariable Cox analysis of OS and CSS in IV CSCLC, SCLC and NSCLC before PSM

|  | | | OS before PSM | | | | | | | | | | | | | | | CSS before PSM | | | | | | | | | | | | | | | |  |
| --- | --- | --- | --- | --- | --- | --- | --- | --- | --- | --- | --- | --- | --- | --- | --- | --- | --- | --- | --- | --- | --- | --- | --- | --- | --- | --- | --- | --- | --- | --- | --- | --- | --- | --- |
|  | | | CSCLC, N =493 | | | | | SCLC, N=35503 | | | | NSCLC,N=122807 | | | | | | | | CSCLC, N =493 | | | | SCLC, N=35503 | | | | | | | NSCLC,N=122807 | | |  |
| Characteristic | | | HR(95%CI) | | | pvalue | | HR(95%CI) | | | pvalue | | | HR(95%CI) | | pvalue | | | HR(95%Cl) | | | pvalue | | | HR(95%CI) | | pvalue | | | HR(95%CI) | | | pvalue |  |
| Age.years. | |  | | |  | |  | |  | | | |  | | |  | | |  | | |  | | |  | |  | |  | | | |  |  |
| ＜65 | |  | | |  | |  | |  | | | | — | | |  | | |  | | |  | | |  | |  | | — | | | |  |  |
| ≥65 | | 1.33(1.09, 1.63) | | | **0.005** | | 1.37(1.34, 1.40) | | **<0.001** | | | | 1.28 (1.26,1.29) | | | **<0.001** | | | 1.29(1.05, 1.59) | | | **0.017** | | | 1.34(1.31, 1.37) | | **<0.001** | | 1.23 (1.21,1.24) | | | | **<0.001** |  |
| Gender | |  | | |  | |  | |  | | | |  | | |  | | |  | | |  | | |  | |  | |  | | | |  |  |
| male | | — | | |  | | — | |  | | | | — | | |  | | | — | | |  | | | — | |  | | — | | | |  |  |
| female | | 0.94(0.78, 1.13) | | | 0.496 | | 0.89(0.87, 0.91) | | **<0.001** | | | | 1.25 (1.24,1.27) | | | **<0.001** | | | 0.95(0.78, 1.15) | | | 0.583 | | | 0.9(0.88, 0.92) | | **<0.001** | | 1.25 (1.23,1.26) | | | | **<0.001** |  |
| Race | |  | | |  | |  | |  | | | |  | | |  | | |  | | |  | | |  | |  | |  | | | |  |  |
| Black | | — | | |  | | — | |  | | | | — | | |  | | | — | | |  | | | — | |  | | — | | | |  |  |
| White | | 1.19(0.90, 1.59) | | | 0.226 | | 1.1(1.06, 1.15) | | **<0.001** | | | | 0.99 (0.97,1.01) | | | 0.353 | | | 1.27(0.93, 1.73) | | | 0.128 | | | 1.13(1.09, 1.18) | | **<0.001** | | 1.01 (0.99 ,1.03) | | | | 0.554 |  |
| Asian or Pacific Islander | | 1.7(1.04, 2.78) | | | **0.036** | | 1.02(0.95, 1.09) | | 0.624 | | | | 0.69 (0.67,0.71) | | | **<0.001** | | | 1.89(1.13, 3.16) | | | **0.015** | | | 1.02(0.95, 1.09) | | 0.650 | | 0.70 (0.68 ,0.72) | | | | **<0.001** |  |
| American Indian/Alaska Native | | | 1.39(0.60, 3.23) | | | 0.447 | | 1.09(0.95, 1.24) | | 0.225 | | | | 1.03 (0.95,1.12) | | | 0.432 | | | 1.61(0.69, 3.78) | | | 0.272 | | | 1.05(0.91, 1.20) | | 0.538 | | 1.04 (0.96 ,1.14) | | | | 0.353 |
| Married.status | |  | | |  | |  | |  | | | |  | | |  | | |  | | |  | | |  | |  | |  | | | |  |  |
| Married | | — | | |  | | — | |  | | | |  | | |  | | | — | | |  | | | — | |  | | — | | | |  |  |
| Divorced | | 1.04(0.79, 1.37) | | | 0.764 | | 1.07(1.03, 1.10) | | **<0.001** | | | | 1.04 (1.02,1.06) | | | **<0.001** | | | 1.08(0.81, 1.43) | | | 0.609 | | | 1.07(1.04, 1.11) | | **<0.001** | | 1.15 (1.12 ,1.17) | | | | **<0.001** |  |
| Others | | 1.01(0.82, 1.24) | | | 0.935 | | 1.13(1.11, 1.16) | | **<0.001** | | | | 0.87 (0.85,0.89) | | | **<0.001** | | | 1.01(0.82, 1.25) | | | 0.903 | | | 1.11(1.09, 1.14) | | **<0.001** | | 1.18 (1.16 ,1.19) | | | | **<0.001** |  |
| Primary.Site | |  | | |  | |  | |  | | | |  | | |  | | |  | | |  | | |  | |  | |  | | | |  |  |
| Main bronchus | | — | | |  | | — | |  | | | | — | | |  | | | — | | |  | | | — | |  | |  | | | |  |  |
| Upper lobe | | 0.82(0.56, 1.19) | | | 0.288 | | 0.99(0.95, 1.02) | | 0.477 | | | | 0.76 (0.73,0.78) | | | **<0.001** | | | 0.77(0.53, 1.13) | | | 0.179 | | | 0.98(0.94, 1.02) | | 0.260 | | 0.75 (0.72,0.77) | | | | **<0.001** |  |
| Middle lobe | | 0.94(0.50, 1.76) | | | 0.844 | | 0.98(0.92, 1.05) | | 0.516 | | | | 0.70 (0.67,0.73) | | | **<0.001** | | | 0.82(0.42, 1.60) | | | 0.563 | | | 0.95(0.89, 1.02) | | 0.165 | | 0.68 (0.65,0.72) | | | | **<0.001** |  |
| Lower lobe | | 0.95(0.64, 1.42) | | | 0.817 | | 1.07(1.03, 1.11) | | **<0.001** | | | | 0.76 (0.74,0.79) | | | **<0.001** | | | 0.9(0.60, 1.35) | | | 0.604 | | | 1.06(1.02, 1.10) | | **0.008** | | 0.76 (0.73,0.78) | | | | **<0.001** |  |
| Others | | 0.83(0.55, 1.25) | | | 0.377 | | 1.17(1.13, 1.22) | | **<0.001** | | | | 0.93(0.90,0.96) | | | **<0.001** | | | 0.78(0.51, 1.18) | | | 0.233 | | | 1.13(1.08, 1.18) | | **<0.001** | | 0.90 (0.87,0.93) | | | | **<0.001** |  |
| Laterality | |  | | |  | |  | |  | | | |  | | |  | | |  | | |  | | |  | |  | |  | | | |  |  |
| Left | | — | | |  | | — | |  | | | | — | | |  | | | — | | |  | | | — | |  | | — | | | |  |  |
| Right | | 1.17(0.96, 1.42) | | | 0.118 | | 0.99(0.96, 1.01) | | 0.266 | | | | 1.01 (0.99,1.02) | | | 0.338 | | | 1.11(0.91, 1.36) | | | 0.302 | | | 0.98(0.96, 1.01) | | 0.157 | | 1.01 (1.00,1.02) | | | | 0.184 |  |
| Others | | 1.12(0.77, 1.62) | | | 0.558 | | 1.14(1.09, 1.19) | | **<0.001** | | | | 1.24 (1.21,1.27) | | | **<0.001** | | | 0.92(0.61, 1.39) | | | 0.706 | | | 1.08(1.03, 1.13) | | **<0.001** | | 1.21 (1.18,1.25) | | | | **<0.001** |  |
| T.stage | |  | | |  | |  | |  | | | |  | | |  | | |  | | |  | | |  | |  | |  | | | |  |  |
| T0 | | — | | |  | | — | |  | | | | — | | |  | | | — | | |  | | | — | |  | | — | | | |  |  |
| T1 | | 0.42(0.16, 1.07) | | | 0.068 | | 1.02(0.92, 1.13) | | 0.733 | | | | 0.77 (0.72,0.82) | | | **<0.001** | | | 0.38(0.15, 0.97) | | | **0.044** | | | 1.08(0.96, 1.20) | | 0.195 | | 0.76 (0.71,0.82) | | | | **<0.001** |  |
| T2 | | 0.42(0.17, 1.04) | | | 0.06 | | 1.16(1.05, 1.29) | | **0.003** | | | | 0.98 (0.92,1.05) | | | **<0.001** | | | 0.38(0.15, 0.93) | | | **0.035** | | | 1.25(1.12, 1.39) | | **<0.001** | | 1.00 (0.93,1.07) | | | | 0.959 |  |
| T3 | | 0.51(0.20, 1.27) | | | 0.149 | | 1.18(1.06, 1.30) | | **0.002** | | | | 1.01 (0.94,1.08) | | | **<0.001** | | | 0.43(0.17, 1.09) | | | 0.074 | | | 1.25(1.12, 1.40) | | **<0.001** | | 1.02 (0.94,1.09) | | | | 0.683 |  |
| T4 | | 0.49(0.20, 1.20) | | | 0.117 | | 1.24(1.13, 1.37) | | **<0.001** | | | | 1.14 (1.07,1.21) | | | **<0.001** | | | 0.45(0.19, 1.11) | | | 0.082 | | | 1.34(1.20, 1.48) | | **<0.001** | | 1.16 (1.09,1.25) | | | | **<0.001** |  |
| TX | | 0.53(0.21, 1.33) | | | 0.176 | | 1.35(1.22, 1.50) | | **<0.001** | | | | 1.22 (1.14,1.30) | | | **<0.001** | | | 0.45(0.18, 1.14) | | | 0.092 | | | 1.42(1.27, 1.58) | | **<0.001** | | 1.22 (1.14,1.31) | | | | **<0.001** |  |
| N.stage | |  | | |  | |  | |  | | | |  | | |  | | |  | | |  | | |  | |  | |  | | | |  |  |
| N0 | | — | | |  | | — | |  | | | | — | | |  | | | — | | |  | | | — | |  | | — | | | |  |  |
| N1 | | 0.9(0.59, 1.38) | | | 0.638 | | 0.94(0.89, 0.99) | | **0.022** | | | | 1.07 (1.04,1.09) | | | **<0.001** | | | 0.91(0.58, 1.43) | | | 0.687 | | | 0.97(0.92, 1.02) | | 0.268 | | 1.10 (1.07,1.13) | | | | **<0.001** |  |
| N2 | | 1.3(1.01, 1.67) | | | **0.045** | | 1.09(1.05, 1.13) | | **<0.001** | | | | 1.24 (1.22,1.26) | | | **<0.001** | | | 1.37(1.05, 1.79) | | | **0.022** | | | 1.12(1.08, 1.17) | | **<0.001** | | 1.29 (1.27,1.31) | | | | **<0.001** |  |
| N3 | | 1.28(0.97, 1.70) | | | 0.083 | | 1.01(0.98, 1.05) | | 0.444 | | | | 1.15 (1.13,1.18) | | | **<0.001** | | | 1.35(1.01, 1.82) | | | **0.045** | | | 1.05(1.01, 1.09) | | **0.026** | | 1.22 (1.19,1.24) | | | | **<0.001** |  |
| NX | | 1.89(1.20, 2.97) | | | **0.006** | | 1.3(1.23, 1.37) | | **<0.001** | | | | 1.39 (1.36,1.43) | | | **<0.001** | | | 1.76(1.07, 2.89) | | | **0.025** | | | 1.29(1.21, 1.36) | | **<0.001** | | 1.41 (1.37,1.45) | | | | **<0.001** |  |
| Bone.Metastasis | |  | | |  | |  | |  | | | |  | | |  | | |  | | |  | | |  | |  | |  | | | |  |  |
| No | | — | | |  | |  | |  | | | |  | | |  | | | — | | |  | | |  | |  | | — | | | |  |  |
| Yes | | 1.31(1.08, 1.60) | | | **0.007** | | 1.09(1.06, 1.11) | | **<0.001** | | | | 1.27 (1.26,1.29) | | | **<0.001** | | | 1.36(1.11, 1.67) | | | **0.003** | | | 1.12(1.09, 1.14) | | **<0.001** | | 1.32 (1.30,1.34) | | | | **<0.001** |  |
| Brain.Metastasis | |  | | |  | |  | |  | | | |  | | |  | | |  | | |  | | |  | |  | |  | | | |  |  |
| No | | — | | |  | | — | |  | | | | — | | |  | | | — | | |  | | | — | |  | | — | | | |  |  |
| Yes | | 1.01(0.82, 1.24) | | | 0.942 | | 0.99(0.96, 1.01) | | 0.331 | | | | 1.06 (1.04,1.07) | | | **<0.001** | | | 1(0.81, 1.24) | | | 0.988 | | | 1.01(0.99, 1.04) | | 0.292 | | 1.10 (1.09,1.12) | | | | **<0.001** |  |
| Liver.Metastasis | |  | | |  | |  | |  | | | |  | | |  | | |  | | |  | | |  | |  | |  | | | |  |  |
| No | | — | | |  | | — | |  | | | | — | | |  | | | — | | |  | | | — | |  | | — | | | |  |  |
| Yes | | 1.47(1.21, 1.80) | | | **<0.001** | | 1.57(1.53, 1.60) | | **<0.001** | | | | 1.56 (1.54,1.59) | | | **<0.001** | | | 1.48(1.20, 1.82) | | | **<0.001** | | | 1.58(1.54, 1.62) | | **<0.001** | | 1.60 (1.58,1.63) | | | | **<0.001** |  |
| Lung.Metastasis | |  | | |  | |  | |  | | | |  | | |  | | |  | | |  | | |  | |  | |  | | | |  |  |
| No | | — | | |  | | — | |  | | | |  | | |  | | | — | | |  | | | — | |  | | — | | | |  |  |
| Yes | | 1.22(0.99, 1.51) | | | 0.061 | | 1.13(1.10, 1.16) | | **<0.001** | | | | 1.04 (1.02,1.05) | | | **<0.001** | | | 1.14(0.91, 1.42) | | | 0.257 | | | 1.13(1.09, 1.16) | | **<0.001** | | 1.04 (1.02,1.05) | | | | **<0.001** |  |
| Surgery | |  | | |  | |  | |  | | | | — | | |  | | |  | | |  | | |  | |  | |  | | | |  |  |
| No | | — | | |  | | — | |  | | | |  | | |  | | | — | | |  | | | — | |  | | — | | | |  |  |
| Yes | | 0.56(0.36, 0.86) | | | **0.009** | | 0.67(0.60, 0.76) | | **<0.001** | | | | 0.46 (0.44,0.47) | | | **<0.001** | | | 0.56(0.36, 0.88) | | | **0.012** | | | 0.67(0.59, 0.76) | | **<0.001** | | 0.44 (0.42,0.46) | | | | **<0.001** |  |
| Radiotherapy | |  | | |  | |  | |  | | | |  | | |  | | |  | | |  | | |  | |  | |  | | | |  |  |
| No | | — | | |  | | — | |  | | | |  | | |  | | | — | | |  | | | — | |  | | — | | | |  |  |
| Yes | | 0.69(0.57, 0.83) | | | **<0.001** | | 0.54(0.53, 0.55) | | **<0.001** | | | | 0.85 (0.84,0.86) | | | **<0.001** | | | 0.71(0.58, 0.86) | | | **<0.001** | | | 0.56(0.55, 0.57) | | **<0.001** | | 0.88 (0.87,0.89) | | | | **<0.001** |  |
| Chemotherapy | |  | | |  | |  | |  | | | |  | | |  | | |  | | |  | | |  | |  | |  | | | |  |  |
| No | | — | | |  | | — | |  | | | | — | | |  | | | — | | |  | | | — | |  | | — | | | |  |  |
| Yes | | 0.34(0.28, 0.41) | | | **<0.001** | | 0.24(0.24, 0.25) | | **<0.001** | | | | 0.41 (0.41,0.42) | | | **<0.001** | | | 0.37(0.30, 0.46) | | | **<0.001** | | | 0.25(0.25, 0.26) | | **<0.001** | | 0.43 (0.42,0.43) | | | | **<0.001** |  |
|  | | |  | |  | |  | |  | |  | | |  | |  | | | |  | |  | | |  | |  | | | |  |  |  |  |
