# Supplementary material for: Clinical features and prognostic factors of IV combined small cell lung cancer: A propensity score matching analysis
Source: PLoS One. 2024 Nov 8;19(11):e0313221. doi: 10.1371/journal.pone.0313221 (PMC11548789; doi:10.1371/journal.pone.0313221)
Supplement: S2 Table — (DOCX) [file pone.0313221.s005.docx]

S2 Table: Univariable Cox analysis of OS and CSS in IV CSCLC, SCLC and NSCLC after 1:4 PSM

|  | | | OS after PSM | | | | | | | | | | | | | | | | CSS after PSM | | | | | | | | | | | | | | | | | |
| --- | --- | --- | --- | --- | --- | --- | --- | --- | --- | --- | --- | --- | --- | --- | --- | --- | --- | --- | --- | --- | --- | --- | --- | --- | --- | --- | --- | --- | --- | --- | --- | --- | --- | --- | --- | --- |
|  | | | CSCLC, N =493 | | | | | SCLC, N=1954 | | | | | | NSCLC, N=1968 | | | | | | CSCLC, N=493 | | | | SCLC, N=1954 | | | | | | | NSCLC, N=1968 | | | | | |
| Characteristic | | | HR(95CI) | | | pvalue | | | HR(95CI) | | | pvalue | | | HR(95CI) | | pvalue | | | HR(95Cl) | | pvalue | | | | HR(95CI) | | | | pvalue | | | | | HR(95CI) | pvalue |
| Age.years. | | |  | | |  | |  | | | | |  | |  | |  | | |  | |  | | |  | | | |  | | |  | | | |  |
| ＜65 | | |  | | |  | |  | | | | |  | |  | |  | | |  | |  | | |  | | | |  | | |  | | | |  |
| ≥65 | | | 1.33(1.09, 1.63) | | | **0.005** | | 1.42(1.29, 1.58) | | | | | **<0.001** | | 1.31(1.19, 1.46) | | **<0.001** | | | 1.29(1.05, 1.59) | | **0.017** | | | 1.4(1.26, 1.56) | | | | **<0.001** | | | 1.27(1.14, 1.42) | | | | **<0.001** |
| Gender | | |  | | |  | |  | | | | |  | |  | |  | | |  | |  | | |  | | | |  | | |  | | | |  |
| male | | | — | | |  | |  | | | | |  | | — | |  | | | — | |  | | | — | | | |  | | | — | | | |  |
| female | | | 0.94(0.78, 1.13) | | | 0.496 | | 0.84(0.77, 0.93) | | | | | **<0.001** | | 0.82(0.75, 0.91) | | **<0.001** | | | 0.95(0.78, 1.15) | | 0.583 | | | 0.85(0.77, 0.94) | | | | **0.001** | | | 0.83(0.75, 0.92) | | | | **<0.001** |
| Race | | |  | | |  | |  | | | | |  | |  | |  | | |  | |  | | |  | | | |  | | |  | | | |  |
| Black | | | — | | |  | |  | | | | |  | | — | |  | | | — | |  | | | — | | | |  | | | — | | | |  |
| White | | | 1.19(0.90, 1.59) | | | 0.226 | | 1.13(0.98, 1.31) | | | | | 0.101 | | 1(0.85, 1.18) | | 0.987 | | | 1.27(0.93, 1.73) | | 0.128 | | | 1.14(0.98, 1.33) | | | | 0.099 | | | 1.03(0.86, 1.23) | | | | 0.755 |
| Asian or Pacific Islander | | | 1.7(1.04, 2.78) | | | **0.036** | | 1.18(0.91, 1.54) | | | | | 0.211 | | 0.66(0.49, 0.88) | | **0.005** | | | 1.89(1.13, 3.16) | | **0.015** | | | 1.14(0.86, 1.51) | | | | 0.365 | | | 0.68(0.50, 0.93) | | | | **0.016** |
| American Indian/Alaska Native | | | 1.39(0.60, 3.23) | | | 0.447 | | 1.26(0.80, 2.00) | | | | | 0.323 | | 1.4(0.79, 2.46) | | 0.245 | | | 1.61(0.69, 3.78) | | 0.272 | | | 1.09(0.66, 1.82) | | | | 0.732 | | | 1.46(0.81, 2.63) | | | | 0.21 |
| Married.status | | |  | | |  | |  | | | | |  | |  | |  | | |  | |  | | |  | | | |  | | |  | | | |  |
| Married | | | — | | |  | |  | | | | |  | | — | |  | | | — | |  | | | — | | | |  | | | — | | | |  |
| Divorced | | | 1.04(0.79, 1.37) | | | 0.764 | | 1.08(0.93, 1.24) | | | | | 0.307 | | 1.12(0.97, 1.28) | | 0.119 | | | 1.08(0.81, 1.43) | | 0.609 | | | 1.09(0.94, 1.27) | | | | 0.238 | | | 1.09(0.94, 1.26) | | | | 0.263 |
| Others | | | 1.01(0.82, 1.24) | | | 0.935 | | 1.3(1.17, 1.44) | | | | | **<0.001** | | 1.15(1.04, 1.28) | | **0.007** | | | 1.01(0.82, 1.25) | | 0.903 | | | 1.27(1.14, 1.41) | | | | **<0.001** | | | 1.12(1.01, 1.25) | | | | **0.039** |
| Primary.Site | | |  | | |  | |  | | | | |  | |  | |  | | |  | |  | | |  | | | |  | | |  | | | |  |
| Main bronchus | | | — | | |  | |  | | | | |  | | — | |  | | | — | |  | | | — | | | |  | | | — | | | |  |
| Upper lobe | | | 0.82(0.56, 1.19) | | | 0.288 | | 0.88(0.73, 1.05) | | | | | 0.161 | | 0.82(0.66, 1.03) | | 0.094 | | | 0.77(0.53, 1.13) | | 0.179 | | | 0.85(0.70, 1.03) | | | | 0.091 | | | 0.82(0.65, 1.04) | | | | 0.104 |
| Middle lobe | | | 0.94(0.50, 1.76) | | | 0.844 | | 0.79(0.57, 1.09) | | | | | 0.153 | | 0.68(0.48, 0.97) | | **0.035** | | | 0.82(0.42, 1.60) | | 0.563 | | | 0.75(0.54, 1.06) | | | | 0.099 | | | 0.68(0.47, 0.98) | | | | **0.040** |
| Lower lobe | | | 0.95(0.64, 1.42) | | | 0.817 | | 0.98(0.80, 1.20) | | | | | 0.847 | | 0.84(0.66, 1.07) | | 0.165 | | | 0.9(0.60, 1.35) | | 0.604 | | | 0.94(0.76, 1.16) | | | | 0.569 | | | 0.83(0.65, 1.07) | | | | 0.151 |
| Others | | | 0.83(0.55, 1.25) | | | 0.377 | | 1.03(0.84, 1.26) | | | | | 0.765 | | 0.91(0.72, 1.16) | | 0.469 | | | 0.78(0.51, 1.18) | | 0.233 | | | 1(0.81, 1.23) | | | | 0.963 | | | 0.91(0.71, 1.17) | | | | 0.465 |
| Laterality | | |  | | |  | |  | | | | |  | |  | |  | | |  | |  | | |  | | | |  | | |  | | | |  |
| Left | | | — | | |  | |  | | | | |  | | — | |  | | | — | |  | | | — | | | |  | | | — | | | |  |
| Right | | | 1.17(0.96, 1.42) | | | 0.118 | | 1.03(0.93, 1.13) | | | | | 0.580 | | 1.01(0.92, 1.12) | | 0.807 | | | 1.11(0.91, 1.36) | | 0.302 | | | 1.02(0.92, 1.13) | | | | 0.695 | | | 0.98(0.88, 1.09) | | | | 0.744 |
| Others | | | 1.12(0.77, 1.62) | | | 0.558 | | 1.03(0.83, 1.27) | | | | | 0.784 | | 1.29(1.08, 1.55) | | **0.005** | | | 0.92(0.61, 1.39) | | 0.706 | | | 1(0.80, 1.25) | | | | 0.995 | | | 1.27(1.05, 1.53) | | | | **0.013** |
| T.stage | | |  | | |  | |  | | | | |  | |  | |  | | |  | |  | | |  | | | |  | | |  | | | |  |
| T0 | | | — | | |  | |  | | | | |  | | — | |  | | | — | |  | | | — | | | |  | | | — | | | |  |
| T1 | | | 0.42(0.16, 1.07) | | | 0.068 | | 1.51(0.84, 2.73) | | | | | 0.171 | | 1.01(0.57, 1.78) | | 0.972 | | | 0.38(0.15, 0.97) | | **0.044** | | | 1.45(0.78, 2.69) | | | | 0.239 | | | 0.91(0.51, 1.60) | | | | 0.734 |
| T2 | | | 0.42(0.17, 1.04) | | | 0.06 | | 1.87(1.05, 3.32) | | | | | **0.032** | | 1.26(0.73, 2.19) | | 0.411 | | | 0.38(0.15, 0.93) | | **0.035** | | | 1.94(1.06, 3.53) | | | | **0.031** | | | 1.17(0.67, 2.03) | | | | 0.584 |
| T3 | | | 0.51(0.20, 1.27) | | | 0.149 | | 1.83(1.02, 3.28) | | | | | **0.042** | | 1.38(0.78, 2.42) | | 0.264 | | | 0.43(0.17, 1.09) | | 0.074 | | | 1.72(0.94, 3.17) | | | | 0.080 | | | 1.22(0.70, 2.16) | | | | 0.483 |
| T4 | | | 0.49(0.20, 1.20) | | | 0.117 | | 1.93(1.09, 3.41) | | | | | **0.024** | | 1.57(0.91, 2.71) | | 0.108 | | | 0.45(0.19, 1.11) | | 0.082 | | | 1.94(1.07, 3.52) | | | | **0.029** | | | 1.41(0.82, 2.45) | | | | 0.217 |
| TX | | 0.53(0.21, 1.33) | | | 0.176 | | 1.87(1.05, 3.36) | | | **0.035** | | | | | 1.83(1.04, 3.21) | | **0.035** | | | 0.45(0.18, 1.14) | | 0.092 | | | 1.84(1.00, 3.39) | | | | **0.050** | | | | 1.6(0.91, 2.82) | | | 0.102 |
| N.stage | |  | | |  | |  | | |  | | | | |  | |  | | |  | |  | | |  | | | |  | | | |  | | |  |
| N0 | | — | | |  | |  | | |  | | | | | — | |  | | | — | |  | | | — | | | |  | | | | — | | |  |
| N1 | | 0.9(0.59, 1.38) | | | 0.638 | | 1.15(0.92, 1.44) | | | 0.208 | | | | | 0.91(0.74, 1.13) | | 0.404 | | | 0.91(0.58, 1.43) | | 0.687 | | | 1.14(0.91, 1.44) | | | | 0.256 | | | | 0.96(0.76, 1.20) | | | 0.698 |
| N2 | | 1.3(1.01, 1.67) | | | **0.045** | | 1.23(1.08, 1.39) | | | **0.002** | | | | | 1.27(1.12, 1.44) | | **<0.001** | | | 1.37(1.05, 1.79) | | **0.022** | | | 1.23(1.07, 1.40) | | | | **0.003** | | | | 1.35(1.18, 1.54) | | | **<0.001** |
| N3 | | 1.28(0.97, 1.70) | | | 0.083 | | 1.06(0.92, 1.23) | | | 0.442 | | | | | 1.25(1.08, 1.45) | | **0.003** | | | 1.35(1.01, 1.82) | | **0.045** | | | 1.07(0.92, 1.24) | | | | 0.416 | | | | 1.3(1.12, 1.52) | | | **<0.001** |
| NX | | 1.89(1.20, 2.97) | | | **0.006** | | 1.3(1.04, 1.62) | | | **0.021** | | | | | 1.73(1.38, 2.17) | | **<0.001** | | | 1.76(1.07, 2.89) | | **0.025** | | | 1.18(0.93, 1.50) | | | | 0.179 | | | | 1.79(1.40, 2.28) | | | **<0.001** |
| Bone.Metastasis | |  | | |  | |  | | |  | | | | |  | |  | | |  | |  | | |  | | | |  | | | |  | | |  |
| No | | — | | |  | |  | | |  | | | | | — | |  | | | — | |  | | | — | | | |  | | | | — | | |  |
| Yes | | 1.31(1.08, 1.60) | | | **0.007** | | 1.2(1.09, 1.33) | | | **<0.001** | | | | | 1.39(1.26, 1.54) | | **<0.001** | | | 1.36(1.11, 1.67) | | **0.003** | | | 1.24(1.12, 1.37) | | | | **<0.001** | | | | 1.39(1.25, 1.54) | | | **<0.001** |
| Brain.Metastasis | |  | | |  | |  | | |  | | | | |  | |  | | |  | |  | | |  | | | |  | | | |  | | |  |
| No | | — | | |  | |  | | |  | | | | | — | |  | | | — | |  | | | — | | | |  | | | | — | | |  |
| Yes | | 1.01(0.82, 1.24) | | | 0.942 | | 0.91(0.82, 1.00) | | | 0.058 | | | | | 1.05(0.95, 1.17) | | 0.317 | | | 1(0.81, 1.24) | | 0.988 | | | 0.93(0.83, 1.03) | | | | 0.170 | | | | 1.09(0.98, 1.22) | | | 0.106 |
| Liver.Metastasis | |  | | |  | |  | | |  | | | | |  | |  | | |  | |  | | |  | | | |  | | | |  | | |  |
| No | | — | | |  | |  | | |  | | | | | — | |  | | | — | |  | | | — | | | |  | | | | — | | |  |
| Yes | | 1.47(1.21, 1.80) | | | **<0.001** | | 1.56(1.41, 1.73) | | | **<0.001** | | | | | 1.43(1.29, 1.59) | | **<0.001** | | | 1.48(1.20, 1.82) | | **<0.001** | | | 1.59(1.43, 1.76) | | | | **<0.001** | | | | 1.45(1.30, 1.61) | | | **<0.001** |
| Lung.Metastasis | |  | | |  | |  | | |  | | | | |  | |  | | |  | |  | | |  | | | |  | | | |  | | |  |
| No | | — | | |  | |  | | |  | | | | | — | |  | | | — | |  | | | — | | | |  | | | | — | | |  |
| Yes | | 1.22(0.99, 1.51) | | | 0.061 | | 1.18(1.06, 1.32) | | | **0.002** | | | | | 1.15(1.03, 1.28) | | **0.012** | | | 1.14(0.91, 1.42) | | 0.257 | | | 1.16(1.04, 1.30) | | | | **0.009** | | | | 1.14(1.01, 1.27) | | | **0.027** |
| Surgery | |  | | |  | |  | | |  | | | | |  | |  | | |  | |  | | |  | | | |  | | | |  | | |  |
| No | | — | | |  | |  | | |  | | | | | — | |  | | | — | |  | | | — | | | |  | | | | — | | |  |
| Yes | | 0.56(0.36, 0.86) | | | **0.009** | | 0.71(0.56, 0.90) | | | **0.005** | | | | | 0.37(0.29, 0.46) | | **<0.001** | | | 0.56(0.36, 0.88) | | **0.012** | | | 0.68(0.53, 0.87) | | | | **0.002** | | | | 0.35(0.28, 0.45) | | | **<0.001** |
| Radiotherapy | |  | | |  | |  | | |  | | | | |  | |  | | |  | |  | | |  | | | |  | | | |  | | |  |
| No | | — | | |  | |  | | |  | | | | | — | |  | | | — | |  | | | — | | | |  | | | | — | | |  |
| Yes | | 0.69(0.57, 0.83) | | | **<0.001** | | 0.54(0.49, 0.59) | | | **<0.001** | | | | | 0.88(0.80, 0.96) | | **0.006** | | | 0.71(0.58, 0.86) | | **<0.001** | | | 0.55(0.50, 0.61) | | | | **<0.001** | | | | 0.87(0.79, 0.96) | | | **0.005** |
| Chemotherapy | |  | | |  | |  | | |  | | | | |  | |  | | |  | |  | | |  | | | |  | | | |  | | |  |
| No | | — | | |  | |  | | |  | | | | | — | |  | | | — | |  | | | — | | | |  | | | | — | | |  |
| Yes | | 0.34(0.28, 0.41) | | | **<0.001** | | 0.25(0.22, 0.27) | | | **<0.001** | | | | | 0.4(0.36, 0.44) | | **<0.001** | | | 0.37(0.30, 0.46) | | **<0.001** | | | 0.26(0.23, 0.29) | | | | **<0.001** | | | | 0.41(0.37, 0.46) | | | **<0.001** |
|  | | |  | |  | |  | | |  | | |  | |  | |  | | |  | |  | | | |  |  | | | | | |  | |  |  |
