# Supplementary material for: Clinical features and prognostic factors of IV combined small cell lung cancer: A propensity score matching analysis
Source: PLoS One. 2024 Nov 8;19(11):e0313221. doi: 10.1371/journal.pone.0313221 (PMC11548789; doi:10.1371/journal.pone.0313221)
Supplement: S3 Table — (DOCX) [file pone.0313221.s006.docx]

S3 Table The baseline data of patients undergoing surgical (No/Yes) intervention for IV CSCLC before and after 1:4 PSM

| **Characteristics** |  | | | **Before PSM** | | |  | | **After 1:4 PSM** | | | | |  |
| --- | --- | --- | --- | --- | --- | --- | --- | --- | --- | --- | --- | --- | --- | --- |
|  | **No**,  N = 468 | | **Yes**,  N = 25 | | **SMD** | **p-value** | **No**,  N = 95 | **Yes**,  N = 25 | | **SMD** | | **p-value** | |  |
| **Age** |  |  | | |  | 0.343 |  |  | | |  | | 0.929 |  |
| ＜65 | 155 (33.1%) | 6 (24.0%) | | | -0.214 |  | 22 (23.2%) | 6 (24.0%) | | | 0.016 | |  |  |
| ≥65 | 313 (66.9%) | 19 (76.0%) | | | 0.214 |  | 73 (76.8%) | 19 (76.0%) | | | -0.016 | |  |  |
| **Gender** |  |  | | |  | 0.128 |  |  | | |  | | 0.694 |  |
| Male | 278 (59.4%) | 11 (44.0%) | | | -0.31 |  | 46 (48.4%) | 11 (44.0%) | | | -0.054 | |  |  |
| Female | 190 (40.6%) | 14 (56.0%) | | | 0.31 |  | 49 (51.6%) | 14 (56.0%) | | | 0.054 | |  |  |
| **Race** |  |  | | |  | 0.934 |  |  | | |  | | 0.526 |  |
| Black | 55 (11.8%) | 2 (8.0%) | | | -0.138 |  | 9 (9.5%) | 2 (8.0%) | | | -0.049 | |  |  |
| White | 383 (81.8%) | 22 (88.0%) | | | 0.19 |  | 85 (89.5%) | 22 (88.0%) | | | -0.051 | |  |  |
| Asian or Pacific Islander | 24 (5.1%) | 1 (4.0%) | | | -0.058 |  | 1 (1.1%) | 1 (4.0%) | | | 0.153 | |  |  |
| American Indian/Alaska Native | | 6 (1.3%) | 0 (0.0%) | | | -0.117 |  | 0 (0.0%) | 0 (0.0%) | | | 0 | |  |
| **Marriedstatus** |  |  | | |  | 0.750 |  |  | | |  | | >0.999 |  |
| Married | 236 (50.4%) | 14 (56.0%) | | | 0.112 |  | 52 (54.7%) | 14 (56.0%) | | | 0.047 | |  |  |
| Divorced | 66 (14.1%) | 4 (16.0%) | | | 0.052 |  | 15 (15.8%) | 4 (16.0%) | | | 0 | |  |  |
| Others | 166 (35.5%) | 7 (28.0%) | | | -0.166 |  | 28 (29.5%) | 7 (28.0%) | | | -0.052 | |  |  |
| **Primarysite** |  |  | | |  | 0.881 |  |  | | |  | | 0.851 |  |
| Main bronchus | 33 (7.1%) | 2 (8.0%) | | | 0.035 |  | 3 (3.2%) | 2 (8.0%) | | | 0.100 | |  |  |
| Upper lobe | 231 (49.4%) | 11 (44.0%) | | | -0.108 |  | 45 (47.4%) | 11 (44.0%) | | | -0.047 | |  |  |
| Middle lobe | 13 (2.8%) | 1 (4.0%) | | | 0.062 |  | 4 (4.2%) | 1 (4.0%) | | | 0 | |  |  |
| Lower lobe | 106 (22.6%) | 6 (24.0%) | | | 0.032 |  | 22 (23.2%) | 6 (24.0%) | | | 0.016 | |  |  |
| Others | 85 (18.2%) | 5 (20.0%) | | | 0.046 |  | 21 (22.1%) | 5 (20.0%) | | | -0.083 | |  |  |
| **Laterality** |  |  | | |  | 0.239 |  |  | | |  | | >0.999 |  |
| Left | 188 (40.2%) | 8 (32.0%) | | | -0.175 |  | 33 (34.7%) | 8 (32.0%) | | | -0.043 | |  |  |
| Right | 244 (52.1%) | 17 (68.0%) | | | 0.34 |  | 62 (65.3%) | 17 (68.0%) | | | 0.043 | |  |  |
| Others | 36 (7.7%) | 0 (0.0%) | | | -0.296 |  | 0 (0.0%) | 0 (0.0%) | | | 0 | |  |  |
| **Tstage** |  |  | | |  | 0.462 |  |  | | |  | | >0.999 |  |
| T0 | 5 (1.1%) | 0 (0.0%) | | | -0.107 |  | 0 (0.0%) | 0 (0.0%) | | | 0 | |  |  |
| T1 | 38 (8.1%) | 3 (12.0%) | | | 0.119 |  | 11 (11.6%) | 3 (12.0%) | | | -0.021 | |  |  |
| T2 | 111 (23.7%) | 10 (40.0%) | | | 0.332 |  | 38 (40.0%) | 10 (40.0%) | | | 0.02 | |  |  |
| T3 | 53 (11.3%) | 2 (8.0%) | | | -0.123 |  | 8 (8.4%) | 2 (8.0%) | | | 0 | |  |  |
| T4 | 212 (45.3%) | 9 (36.0%) | | | -0.194 |  | 32 (33.7%) | 9 (36.0%) | | | 0.035 | |  |  |
| TX | 49 (10.5%) | 1 (4.0%) | | | -0.33 |  | 6 (6.3%) | 1 (4.0%) | | | -0.102 | |  |  |
| **Nstage** |  |  | | |  | <0.001 |  |  | | |  | | >0.999 |  |
| N0 | 85 (18.2%) | 10 (40.0%) | | | 0.446 |  | 38 (40.0%) | 10 (40.0%) | | | -0.007 | |  |  |
| N1 | 28 (6.0%) | 5 (20.0%) | | | 0.35 |  | 19 (20.0%) | 5 (20.0%) | | | -0.033 | |  |  |
| N2 | 214 (45.7%) | 5 (20.0%) | | | -0.643 |  | 19 (20.0%) | 5 (20.0%) | | | 0.025 | |  |  |
| N3 | 118 (25.2%) | 3 (12.0%) | | | -0.407 |  | 10 (10.5%) | 3 (12.0%) | | | 0.062 | |  |  |
| NX | 23 (4.9%) | 2 (8.0%) | | | 0.114 |  | 9 (9.5%) | 2 (8.0%) | | | -0.049 | |  |  |
| **BoneMetastasis** |  |  | | |  | 0.266 |  |  | | |  | | 0.589 |  |
| Yes | 163 (34.8%) | 6 (24.0%) | | | -0.254 |  | 28 (29.5%) | 6 (24.0%) | | | -0.094 | |  |  |
| No | 305 (65.2%) | 19 (76.0%) | | | 0.254 |  | 67 (70.5%) | 19 (76.0%) | | | 0.094 | |  |  |
| **BrainMetastasis** |  |  | | |  | 0.062 |  |  | | |  | | >0.999 |  |
| Yes | 137 (29.3%) | 3 (12.0%) | | | -0.532 |  | 12 (12.6%) | 3 (12.0%) | | | 0 | |  |  |
| No | 331 (70.7%) | 22 (88.0%) | | | 0.532 |  | 83 (87.4%) | 22 (88.0%) | | | 0 | |  |  |
| **LiverMetastasis** |  |  | | |  | 0.029 |  |  | | |  | | 0.733 |  |
| Yes | 154 (32.9%) | 3 (12.0%) | | | -0.643 |  | 10 (10.5%) | 3 (12.0%) | | | 0.062 | |  |  |
| No | 314 (67.1%) | 22 (88.0%) | | | 0.643 |  | 85 (89.5%) | 22 (88.0%) | | | -0.062 | |  |  |
| **LungMetastasis** |  |  | | |  | 0.855 |  |  | | |  | | 0.661 |  |
| Yes | 120 (25.6%) | 6 (24.0%) | | | -0.038 |  | 19 (20.0%) | 6 (24.0%) | | | 0.055 | |  |  |
| No | 348 (74.4%) | 19 (76.0%) | | | 0.038 |  | 76 (80.0%) | 19 (76.0%) | | | -0.055 | |  |  |
| **Radiotherapy** |  |  | | |  | 0.246 |  |  | | |  | | 0.968 |  |
| Yes | 205 (43.8%) | 8 (32.0%) | | | -0.253 |  | 30 (31.6%) | 8 (32.0%) | | | 0.021 | |  |  |
| No | 263 (56.2%) | 17 (68.0%) | | | 0.253 |  | 65 (68.4%) | 17 (68.0%) | | | -0.021 | |  |  |
| **Chemotherapy** |  |  | | |  | 0.992 |  |  | | |  | | 0.518 |  |
| Yes | 300 (64.1%) | 16 (64.0%) | | | -0.002 |  | 54 (56.8%) | 16 (64.0%) | | | 0.08 | |  |  |
| No | 168 (35.9%) | 9 (36.0%) | | | 0.002 |  | 41 (43.2%) | 9 (36.0%) | | | -0.08 | |  |  |
