# Supplementary material for: Clinical features and prognostic factors of IV combined small cell lung cancer: A propensity score matching analysis
Source: PLoS One. 2024 Nov 8;19(11):e0313221. doi: 10.1371/journal.pone.0313221 (PMC11548789; doi:10.1371/journal.pone.0313221)
Supplement: S4 Table — (DOCX) [file pone.0313221.s007.docx]

S4 Table The baseline data of patients undergoing radiotherapy (No/Yes) intervention for IV CSCLC before and after PSM

| **Characteristics** | |  | | | **Before PSM** | | |  | | **After 1:1 PSM** | | | | |  |
| --- | --- | --- | --- | --- | --- | --- | --- | --- | --- | --- | --- | --- | --- | --- | --- |
|  |  | **No**,  N = 280 | | **Yes**,  N = 213 | | **SMD** | **p-value** | **No**,  N = 129 | **Yes**,  N = 129 | | **SMD** | | **p-value** | |  |
| **Age** | |  |  | | |  | 0.043 |  |  | | |  | | >0.999 |  |
| ＜65 | | 81 (28.9%) | 80 (37.6%) | | | 0.178 |  | 47 (36.4%) | 47 (36.4%) | | | 0 | |  |  |
| ≥65 | | 199 (71.1%) | 133 (62.4%) | | | -0.178 |  | 82 (63.6%) | 82 (63.6%) | | | 0 | |  |  |
| **Gender** | |  |  | | |  | 0.257 |  |  | | |  | | >0.999 |  |
| Male | | 158 (56.4%) | 131 (61.5%) | | | 0.104 |  | 77 (59.7%) | 77 (59.7%) | | | 0 | |  |  |
| Female | | 122 (43.6%) | 82 (38.5%) | | | -0.104 |  | 52 (40.3%) | 52 (40.3%) | | | 0 | |  |  |
| **Race** | |  |  | | |  | 0.395 |  |  | | |  | | >0.999 |  |
| Black | | 38 (13.6%) | 19 (8.9%) | | | -0.163 |  | 14 (10.9%) | 14 (10.9%) | | | 0 | |  |  |
| White | | 224 (80.0%) | 181 (85.0%) | | | 0.139 |  | 107 (82.9%) | 107 (82.9%) | | | 0 | |  |  |
| Asian or Pacific Islander | | 15 (5.4%) | 10 (4.7%) | | | -0.031 |  | 7 (5.4%) | 7 (5.4%) | | | 0 | |  |  |
| American Indian/Alaska Native | | | 3 (1.1%) | 3 (1.4%) | | | 0.029 |  | 1 (0.8%) | 1 (0.8%) | | | 0 | |  |
| **Married status** | |  |  | | |  | 0.674 |  |  | | |  | | 0.732 |  |
| Married | | 141 (50.4%) | 109 (51.2%) | | | 0.016 |  | 60 (46.5%) | 60 (46.5%) | | | 0 | |  |  |
| Divorced | | 37 (13.2%) | 33 (15.5%) | | | 0.063 |  | 15 (11.6%) | 19 (14.7%) | | | 0.086 | |  |  |
| Others | | 102 (36.4%) | 71 (33.3%) | | | -0.066 |  | 54 (41.9%) | 50 (38.8%) | | | -0.066 | |  |  |
| **Primary site** | |  |  | | |  | 0.045 |  |  | | |  | | 0.930 |  |
| Main bronchus | | 20 (7.1%) | 15 (7.0%) | | | -0.004 |  | 7 (5.4%) | 7 (5.4%) | | | 0 | |  |  |
| Upper lobe | | 125 (44.6%) | 117 (54.9%) | | | 0.207 |  | 65 (50.4%) | 67 (51.9%) | | | 0.031 | |  |  |
| Middle lobe | | 11 (3.9%) | 3 (1.4%) | | | -0.214 |  | 2 (1.6%) | 3 (2.3%) | | | 0.066 | |  |  |
| Lower lobe | | 63 (22.5%) | 49 (23.0%) | | | 0.012 |  | 29 (22.5%) | 31 (24.0%) | | | 0.037 | |  |  |
| Others | | 61 (21.8%) | 29 (13.6%) | | | -0.238 |  | 26 (20.2%) | 21 (16.3%) | | | -0.100 | |  |  |
| **Laterality** | |  |  | | |  | 0.269 |  |  | | |  | | 0.946 |  |
| Left | | 111 (39.6%) | 85 (39.9%) | | | 0.005 |  | 53 (41.1%) | 55 (42.6%) | | | 0.032 | |  |  |
| Right | | 144 (51.4%) | 117 (54.9%) | | | 0.07 |  | 68 (52.7%) | 67 (51.9%) | | | -0.016 | |  |  |
| Others | | 25 (8.9%) | 11 (5.2%) | | | -0.17 |  | 8 (6.2%) | 7 (5.4%) | | | -0.035 | |  |  |
| **T stage** | |  |  | | |  | 0.482 |  |  | | |  | | 0.959 |  |
| T0 | | 2 (0.7%) | 3 (1.4%) | | | 0.059 |  | 2 (1.6%) | 1 (0.8%) | | | -0.066 | |  |  |
| T1 | | 22 (7.9%) | 19 (8.9%) | | | 0.037 |  | 11 (8.5%) | 12 (9.3%) | | | 0.027 | |  |  |
| T2 | | 65 (23.2%) | 56 (26.3%) | | | 0.07 |  | 27 (20.9%) | 30 (23.3%) | | | 0.053 | |  |  |
| T3 | | 34 (12.1%) | 21 (9.9%) | | | -0.077 |  | 15 (11.6%) | 13 (10.1%) | | | -0.052 | |  |  |
| T4 | | 123 (43.9%) | 98 (46.0%) | | | 0.042 |  | 61 (47.3%) | 63 (48.8%) | | | 0.031 | |  |  |
| TX | | 34 (12.1%) | 16 (7.5%) | | | -0.176 |  | 13 (10.1%) | 10 (7.8%) | | | -0.088 | |  |  |
| **N stage** | |  |  | | |  | 0.811 |  |  | | |  | | 0.896 |  |
| N0 | | 52 (18.6%) | 43 (20.2%) | | | 0.04 |  | 22 (17.1%) | 20 (15.5%) | | | -0.039 | |  |  |
| N1 | | 18 (6.4%) | 15 (7.0%) | | | 0.024 |  | 9 (7.0%) | 11 (8.5%) | | | 0.061 | |  |  |
| N2 | | 121 (43.2%) | 98 (46.0%) | | | 0.056 |  | 61 (47.3%) | 58 (45.0%) | | | -0.047 | |  |  |
| N3 | 73 (26.1%) | | 48 (22.5%) | | | -0.085 |  | 30 (23.3%) | 35 (27.1%) | | | 0.093 | |  |  |
| NX | 16 (5.7%) | | 9 (4.2%) | | | -0.074 |  | 7 (5.4%) | 5 (3.9%) | | | -0.077 | |  |  |
| **Bone Metastasis** |  | |  | | |  | 0.181 |  |  | | |  | | 0.899 |  |
| Yes | 89 (31.8%) | | 80 (37.6%) | | | 0.119 |  | 52 (40.3%) | 51 (39.5%) | | | -0.016 | |  |  |
| No | 191 (68.2%) | | 133 (62.4%) | | | -0.119 |  | 77 (59.7%) | 78 (60.5%) | | | 0.016 | |  |  |
| **Brain Metastasis** |  | |  | | |  | <0.001 |  |  | | |  | | 0.664 |  |
| Yes | 30 (10.7%) | | 110 (51.6%) | | | 0.819 |  | 30 (23.3%) | 33 (25.6%) | | | 0.047 | |  |  |
| No | 250 (89.3%) | | 103 (48.4%) | | | -0.819 |  | 99 (76.7%) | 96 (74.4%) | | | -0.047 | |  |  |
| **Liver Metastasis** |  | |  | | |  | 0.007 |  |  | | |  | | 0.050 |  |
| Yes | 103 (36.8%) | | 54 (25.4%) | | | -0.263 |  | 28 (21.7%) | 42 (32.6%) | | | 0.088 | |  |  |
| No | 177 (63.2%) | | 159 (74.6%) | | | 0.263 |  | 101 (78.3%) | 87 (67.4%) | | | -0.089 | |  |  |
| **Lung Metastasis** |  | |  | | |  | 0.030 |  |  | | |  | | 0.885 |  |
| Yes | 82 (29.3%) | | 44 (20.7%) | | | -0.213 |  | 32 (24.8%) | 31 (24.0%) | | | -0.019 | |  |  |
| No | 198 (70.7%) | | 169 (79.3%) | | | 0.213 |  | 97 (75.2%) | 98 (76.0%) | | | 0.019 | |  |  |
| **surgery** |  | |  | | |  | 0.246 |  |  | | |  | | 0.722 |  |
| Yes | 17 (6.1%) | | 8 (3.8%) | | | -0.122 |  | 3 (2.3%) | 5 (3.9%) | | | 0.082 | |  |  |
| No | 263 (93.9%) | | 205 (96.2%) | | | 0.122 |  | 126 (97.7%) | 124 (96.1%) | | | -0.082 | |  |  |
| **Chemotherapy** |  | |  | | |  | <0.001 |  |  | | |  | | 0.590 |  |
| Yes | 153 (54.6%) | | 163 (76.5%) | | | 0.516 |  | 87 (67.4%) | 91 (70.5%) | | | 0.073 | |  |  |
| No | 127 (45.4%) | | 50 (23.5%) | | | -0.516 |  | 42 (32.6%) | 38 (29.5%) | | | -0.073 | |  |  |
