# Supplementary material for: Clinical features and prognostic factors of IV combined small cell lung cancer: A propensity score matching analysis
Source: PLoS One. 2024 Nov 8;19(11):e0313221. doi: 10.1371/journal.pone.0313221 (PMC11548789; doi:10.1371/journal.pone.0313221)
Supplement: S5 Table — (DOCX) [file pone.0313221.s008.docx]

S5 Table The baseline data of patients undergoing chemotherapy(No/Yes) intervention for IV CSCLC before and after PSM

| **Characteristics** | |  | | | | **Before PSM** | | |  | | **After 1:1 PSM** | | | | |  |
| --- | --- | --- | --- | --- | --- | --- | --- | --- | --- | --- | --- | --- | --- | --- | --- | --- |
|  |  | **No**,  N =177 | | | **Yes**,  N = 316 | | **SMD** | **p-value** | **No**,  N = 157 | **Yes**,  N = 157 | | **SMD** | | **p-value** | |  |
| **Age** | |  | |  | | |  | 0.010 |  |  | | |  | | 0.701 |  |
| ＜65 | | 45 (25.4%) | | 116 (36.7%) | | | 0.259 |  | 40 (25.5%) | 43 (27.4%) | | | 0.044 | |  |  |
| ≥65 | | 132 (74.6%) | | 200 (63.3%) | | | 0.259 |  | 117 (74.5%) | 114 (72.6%) | | | 0.044 | |  |  |
| **Gender** | |  | |  | | |  | 0.669 |  |  | | |  | | 0.172 |  |
| Male | | 106 (59.9%) | | 183 (57.9%) | | | 0.04 |  | 95 (60.5%) | 83 (52.9%) | | | 0.099 | |  |  |
| Female | | 71 (40.1%) | | 133 (42.1%) | | | -0.04 |  | 62 (39.5%) | 74 (47.1%) | | | -0.100 | |  |  |
| **Race** | |  | |  | | |  | 0.091 |  |  | | |  | | 0.934 |  |
| Black | | 23 (13.0%) | | 34 (10.8%) | | | 0.066 |  | 20 (12.7%) | 22 (14.0%) | | | -0.038 | |  |  |
| White | | 140 (79.1%) | | 265 (83.9%) | | | -0.117 |  | 128 (81.5%) | 128 (81.5%) | | | 0 | |  |  |
| Asian or Pacific Islander | | 9 (5.1%) | | 16 (5.1%) | | | 0.001 |  | 8 (5.1%) | 6 (3.8%) | | | 0.058 | |  |  |
| American Indian/Alaska Native | | | | 5 (2.8%) | 1 (0.3%) | | | 0.151 |  | 1 (0.6%) | 1 (0.6%) | | | 0 | |  |
| **Married status** | |  | |  | | |  | 0.017 |  |  | | |  | | 0.848 |  |
| Married | | 76 (42.9%) | | 174 (55.1%) | | | -0.245 |  | 72 (45.9%) | 73 (46.5%) | | | -0.013 | |  |  |
| Divorced | | 25 (14.1%) | | 45 (14.2%) | | | -0.003 |  | 22 (14.0%) | 25 (15.9%) | | | -0.055 | |  |  |
| Others | | 76 (42.9%) | | 97 (30.7%) | | | 0.247 |  | 63 (40.1%) | 59 (37.6%) | | | 0.051 | |  |  |
| **Primary site** | |  | |  | | |  | 0.155 |  |  | | |  | | 0.962 |  |
| Main bronchus | | 11 (6.2%) | | 24 (7.6%) | | | -0.057 |  | 11 (7.0%) | 8 (5.1%) | | | 0.079 | |  |  |
| Upper lobe | | 77 (43.5%) | | 165 (52.2%) | | | -0.176 |  | 70 (44.6%) | 72 (45.9%) | | | -0.026 | |  |  |
| Middle lobe | | 8 (4.5%) | | 6 (1.9%) | | | 0.126 |  | 7 (4.5%) | 6 (3.8%) | | | 0.031 | |  |  |
| Lower lobe | | 43 (24.3%) | | 69 (21.8%) | | | 0.057 |  | 37 (23.6%) | 38 (24.2%) | | | -0.015 | |  |  |
| Others | | 38 (21.5%) | | 52 (16.5%) | | | 0.122 |  | 32 (20.4%) | 33 (21.0%) | | | -0.016 | |  |  |
| **Laterality** | |  | |  | | |  | 0.154 |  |  | | |  | | 0.962 |  |
| Left | | 61 (34.5%) | | 135 (42.7%) | | | -0.174 |  | 60 (38.2%) | 58 (36.9%) | | | 0.027 | |  |  |
| Right | | 100 (56.5%) | | 161 (50.9%) | | | 0.112 |  | 84 (53.5%) | 85 (54.1%) | | | -0.013 | |  |  |
| Others | | 16 (9.0%) | | 20 (6.3%) | | | 0.095 |  | 13 (8.3%) | 14 (8.9%) | | | -0.022 | |  |  |
| **T stage** | |  | |  | | |  | 0.243 |  |  | | |  | | 0.912 |  |
| T0 | | 4 (2.3%) | | 1 (0.3%) | | | 0.131 |  | 1 (0.6%) | 1 (0.6%) | | | 0 | |  |  |
| T1 | | 14 (7.9%) | | 27 (8.5%) | | | -0.024 |  | 14 (8.9%) | 13 (8.3%) | | | 0.024 | |  |  |
| T2 | | 38 (21.5%) | | 83 (26.3%) | | | -0.117 |  | 35 (22.3%) | 35 (22.3%) | | | 0 | |  |  |
| T3 | | 21 (11.9%) | | 34 (10.8%) | | | 0.034 |  | 19 (12.1%) | 16 (10.2%) | | | 0.059 | |  |  |
| T4 | | 78 (44.1%) | | 143 (45.3%) | | | -0.024 |  | 70 (44.6%) | 67 (42.7%) | | | 0.038 | |  |  |
| TX | | 22 (12.4%) | | 28 (8.9%) | | | 0.108 |  | 18 (11.5%) | 25 (15.9%) | | | -0.099 | |  |  |
| **N stage** | |  | |  | | |  | 0.391 |  |  | | |  | | 0.829 |  |
| N0 | | 40 (22.6%) | | 55 (17.4%) | | | 0.124 |  | 32 (20.4%) | 30 (19.1%) | | | 0.03 | |  |  |
| N1 | | 11 (6.2%) | | 22 (7.0%) | | | -0.031 |  | 11 (7.0%) | 7 (4.5%) | | | 0.100 | |  |  |
| N2 | | 73 (41.2%) | | 146 (46.2%) | | | -0.101 |  | 66 (42.0%) | 72 (45.9%) | | | -0.078 | |  |  |
| N3 | 41 (23.2%) | | | 80 (25.3%) | | | -0.051 |  | 38 (24.2%) | 40 (25.5%) | | | -0.03 | |  |  |
| NX | 12 (6.8%) | | | 13 (4.1%) | | | 0.106 |  | 10 (6.4%) | 8 (5.1%) | | | 0.051 | |  |  |
| **Bone Metastasis** |  | | |  | | |  | 0.035 |  |  | | |  | | 0.400 |  |
| Yes | 50 (28.2%) | | | 119 (37.7%) | | | -0.209 |  | 48 (30.6%) | 55 (35.0%) | | | -0.099 | |  |  |
| No | 127 (71.8%) | | | 197 (62.3%) | | | 0.209 |  | 109 (69.4%) | 102 (65.0%) | | | 0.099 | |  |  |
| **Brain Metastasis** |  | | |  | | |  | 0.130 |  |  | | |  | | 0.591 |  |
| Yes | 43 (24.3%) | | | 97 (30.7%) | | | -0.149 |  | 38 (24.2%) | 34 (21.7%) | | | 0.059 | |  |  |
| No | 134 (75.7%) | | | 219 (69.3%) | | | 0.149 |  | 119 (75.8%) | 123 (78.3%) | | | -0.059 | |  |  |
| **Liver Metastasis** |  | | |  | | |  | 0.633 |  |  | | |  | | 0.805 |  |
| Yes | 54 (30.5%) | | | 103 (32.6%) | | | -0.045 |  | 48 (30.6%) | 46 (29.3%) | | | 0.028 | |  |  |
| No | 123 (69.5%) | | | 213 (67.4%) | | | 0.045 |  | 109 (69.4%) | 111 (70.7%) | | | -0.028 | |  |  |
| **Lung Metastasis** |  | | |  | | |  | 0.095 |  |  | | |  | | 0.902 |  |
| Yes | 53 (29.9%) | | | 73 (23.1%) | | | 0.149 |  | 47 (29.9%) | 48 (30.6%) | | | -0.014 | |  |  |
| No | 124 (70.1%) | | | 243 (76.9%) | | | -0.149 |  | 110 (70.1%) | 109 (69.4%) | | | 0.014 | |  |  |
| **surgery** |  | | |  | | |  | 0.992 |  |  | | |  | | >0.999 |  |
| Yes | 9 (5.1%) | | | 16 (5.1%) | | | 0.001 |  | 8 (5.1%) | 8 (5.1%) | | | 0 | |  |  |
| No | 168 (94.9%) | | | 300 (94.9%) | | | -0.001 |  | 149 (94.9%) | 149 (94.9%) | | | 0 | |  |  |
| **Radiotherapy** |  | | |  | | |  | <0.001 |  |  | | |  | | 0.534 |  |
| Yes | 50 (28.2%) | | | 163 (51.6%) | | | -0.518 |  | 48 (30.6%) | 43 (27.4%) | | | 0.071 | |  |  |
| No | 127 (71.8%) | | | 153 (48.4%) | | | 0.518 |  | 109 (69.4%) | 114 (72.6%) | | | -0.071 | |  |  |
