# Supplementary material for: Clinical features and prognostic factors of IV combined small cell lung cancer: A propensity score matching analysis
Source: PLoS One. 2024 Nov 8;19(11):e0313221. doi: 10.1371/journal.pone.0313221 (PMC11548789; doi:10.1371/journal.pone.0313221)
Supplement: S6 Table — (DOCX) [file pone.0313221.s009.docx]

S6 Table. The baseline characteristics of different treatment modalities in IV CSCLC

| **Characteristic** | **Group** | | | | | | | | | **p-value** |  |  |
| --- | --- | --- | --- | --- | --- | --- | --- | --- | --- | --- | --- | --- |
|  | **Control,**  **N = 118** | **Surgery,**  **N = 9** | **Chemotherapy,**  **N = 145** | **Radiotherapy,**  **N = 50** | **Chemoradiotherapy**  **N = 155** | **Surgery+**  **chemotherapy,**  **N = 8** | **Surgery+ chemoradiotherapy,**  **N = 8** | | |  |  |  |
| **Age(years)** |  |  |  |  |  |  | |  | 0.040 | |  |  |
| ＜65 | 32 (27.1) | 1 (11.1) | 47 (32.4) | 12 (24.0) | 64 (41.3) | 1 (12.5) | | 4 (50.0) |  | |  |  |
| ≥65 | 86 (72.9) | 8 (88.9) | 98 (67.6) | 38 (76.0) | 91 (58.7) | 7 (87.5) | | 4 (50.0) |  | |  |  |
| **Gender** |  |  |  |  |  |  | |  | 0.289 | |  |  |
| male | 73 (61.9) | 2 (22.2) | 79 (54.5) | 31 (62.0) | 95 (61.3) | 4 (50.0) | | 5 (62.5) |  | |  |  |
| female | 45 (38.1) | 7 (77.8) | 66 (45.5) | 19 (38.0) | 60 (38.7) | 4 (50.0) | | 3 (37.5) |  | |  |  |
| **Race** |  |  |  |  |  |  | |  | 0.697 | |  |  |
| Black | 17 (14.4) | 1 (11.1) | 20 (13.8) | 5 (10.0) | 13 (8.4) | 0 (0.0) | | 1 (12.5) |  | |  |  |
| White | 92 (78.0) | 7 (77.8) | 117 (80.7) | 41 (82.0) | 133 (85.8) | 8 (100.0) | | 7 (87.5) |  | |  |  |
| Asian or Pacific Islander | | 7 (5.9) | 1 (11.1) | 8 (5.5) | 2 (4.0) | 8 (5.2) | 0 (0.0) | | 0 (0.0) |  | |  |
| American Indian/Alaska Native | | | 2 (1.7) | 0 (0.0) | 0 (0.0) | 2 (4.0) | 1 (0.6) | 0 (0.0) | | 0 (0.0) |  | |
| **Married status** |  |  |  |  |  |  | |  | 0.117 | |  |  |
| Married | 52 (44.1) | 2 (22.2) | 82 (56.6) | 22 (44.0) | 80 (51.6) | 5 (62.5) | | 7 (87.5) |  | |  |  |
| Divorced | 14 (11.9) | 2 (22.2) | 19 (13.1) | 9 (18.0) | 24 (15.5) | 2 (25.0) | | 0 (0.0) |  | |  |  |
| Others | 52 (44.1) | 5 (55.6) | 44 (30.3) | 19 (38.0) | 51 (32.9) | 1 (12.5) | | 1 (12.5) |  | |  |  |
| **Primary Site** |  |  |  |  |  |  | |  | 0.265 | |  |  |
| Main bronchus | 8 (6.8) | 0 (0.0) | 12 (8.3) | 3 (6.0) | 11 (7.1) | 1 (12.5) | | 1 (12.5) |  | |  |  |
| Upper lobe | 50 (42.4) | 2 (22.2) | 69 (47.6) | 25 (50.0) | 87 (56.1) | 4 (50.0) | | 5 (62.5) |  | |  |  |
| Middle lobe | 7 (5.9) | 1 (11.1) | 3 (2.1) | 0 (0.0) | 3 (1.9) | 0 (0.0) | | 0 (0.0) |  | |  |  |
| Lower lobe | 24 (20.3) | 4 (44.4) | 33 (22.8) | 15 (30.0) | 34 (21.9) | 2 (25.0) | | 0 (0.0) |  | |  |  |
| Others | 29 (24.6) | 2 (22.2) | 28 (19.3) | 7 (14.0) | 20 (12.9) | 1 (12.5) | | 2 (25.0) |  | |  |  |
| **Laterality** |  |  |  |  |  |  | |  | 0.378 | |  |  |
| Left | 38 (32.2) | 2 (22.2) | 68 (46.9) | 21 (42.0) | 61 (39.4) | 3 (37.5) | | 3 (37.5) |  | |  |  |
| Right | 67 (56.8) | 7 (77.8) | 65 (44.8) | 27 (54.0) | 86 (55.5) | 5 (62.5) | | 5 (62.5) |  | |  |  |
| Others | 13 (11.0) | 0 (0.0) | 12 (8.3) | 2 (4.0) | 8 (5.2) | 0 (0.0) | | 0 (0.0) |  | |  |  |
| **T stage** |  |  |  |  |  |  | |  | 0.603 | |  |  |
| T0 | 2 (1.7) | 0 (0.0) | 0 (0.0) | 2 (4.0) | 1 (0.6) | 0 (0.0) | | 0 (0.0) |  | |  |  |
| T1 | 8 (6.8) | 2 (22.2) | 11 (7.6) | 4 (8.0) | 15 (9.7) | 1 (12.5) | | 0 (0.0) |  | |  |  |
| T2 | 25 (21.2) | 1 (11.1) | 35 (24.1) | 12 (24.0) | 39 (25.2) | 4 (50.0) | | 5 (62.5) |  | |  |  |
| T3 | 14 (11.9) | 2 (22.2) | 18 (12.4) | 5 (10.0) | 16 (10.3) | 0 (0.0) | | 0 (0.0) |  | |  |  |
| T4 | 52 (44.1) | 3 (33.3) | 65 (44.8) | 23 (46.0) | 72 (46.5) | 3 (37.5) | | 3 (37.5) |  | |  |  |
| TX | 17 (14.4) | 1 (11.1) | 16 (11.0) | 4 (8.0) | 12 (7.7) | 0 (0.0) | | 0 (0.0) |  | |  |  |
| **N stage** |  |  |  |  |  |  | |  | 0.017 | |  |  |
| N0 | 24 (20.3) | 3 (33.3) | 21 (14.5) | 13 (26.0) | 27 (17.4) | 4 (50.0) | | 3 (37.5) |  | |  |  |
| N1 | 8 (6.8) | 1 (11.1) | 9 (6.2) | 2 (4.0) | 9 (5.8) | 0 (0.0) | | 4 (50.0) |  | |  |  |
| N2 | 45 (38.1) | 3 (33.3) | 71 (49.0) | 25 (50.0) | 73 (47.1) | 2 (25.0) | | 0 (0.0) |  | |  |  |
| N3 | 32 (27.1) | 1 (11.1) | 39 (26.9) | 8 (16.0) | 39 (25.2) | 1 (12.5) | | 1 (12.5) |  | |  |  |
| NX | 9 (7.6) | 1 (11.1) | 5 (3.4) | 2 (4.0) | 7 (4.5) | 1 (12.5) | | 0 (0.0) |  | |  |  |
| **Bone Metastasis** |  |  |  |  |  |  | |  | 0.121 | |  |  |
| Yes | 29 (24.6) | 2 (22.2) | 55 (37.9) | 19 (38.0) | 60 (38.7) | 3 (37.5) | | 1 (12.5) |  | |  |  |
| No | 89 (75.4) | 7 (77.8) | 90 (62.1) | 31 (62.0) | 95 (61.3) | 5 (62.5) | | 7 (87.5) |  | |  |  |
| **Brain Metastasis** |  |  |  |  |  |  | |  | ＜0.001 | |  |  |
| Yes | 16 (13.6) | 0 (0.0) | 14 (9.7) | 27 (54.0) | 80 (51.6) | 0 (0.0) | | 3 (37.5) |  | |  |  |
| No | 102 (86.4) | 9 (100.0) | 131 (90.3) | 23 (46.0) | 75 (48.4) | 8 (100.0) | | 5 (62.5) |  | |  |  |
| **Liver Metastasis** |  |  |  |  |  |  | |  | 0.014 | |  |  |
| Yes | 45 (38.1) | 1 (11.1) | 56 (38.6) | 8 (16.0) | 45 (29.0) | 1 (12.5) | | 1 (12.5) |  | |  |  |
| No | 73 (61.9) | 8 (88.9) | 89 (61.4) | 42 (84.0) | 110 (71.0) | 7 (87.5) | | 7 (87.5) |  | |  |  |
| **Lung Metastasis** |  |  |  |  |  |  | |  | 0.226 | |  |  |
| Yes | 37 (31.4) | 2 (22.2) | 40 (27.6) | 14 (28.0) | 29 (18.7) | 3 (37.5) | | 1 (12.5) |  | |  |  |
| No | 81 (68.6) | 7 (77.8) | 105 (72.4) | 36 (72.0) | 126 (81.3) | 5 (62.5) | | 7 (87.5) |  | |  |  |
|  | | | | | | | | | | |  |  |
