# Supplementary material for: Clinical features and prognostic factors of IV combined small cell lung cancer: A propensity score matching analysis
Source: PLoS One. 2024 Nov 8;19(11):e0313221. doi: 10.1371/journal.pone.0313221 (PMC11548789; doi:10.1371/journal.pone.0313221)
Supplement: S7 Table — (DOCX) [file pone.0313221.s010.docx]

S7 Table. The baseline characteristics of different treatment modalities in IV SCLC

| **Characteristic** | **Group** | | | | | | | | | | |  |  |  |
| --- | --- | --- | --- | --- | --- | --- | --- | --- | --- | --- | --- | --- | --- | --- |
|  | **Control**  **N = 9,490** | | **Surgery**  **N = 88** | **Chemotherapy**  **N = 12,550** | **Radiotherapy, N = 2,185** | **Chemoradiotherapy**  **N = 10,949** | **Surgery**  **+chemotherapy**  **N = 116** | **Surgery +radiotherapy**  **N = 17** | **Surgery+ chemoradiotherapy**  **N = 108** | | **p-value** | |  |  |
| **Age(years)** |  | |  |  |  |  |  |  |  | <0.001 | | |  |  |
| ＜65 | 2,265 (23.9) | | 33 (37.5) | 4,664 (37.2) | 685 (31.4) | 5,261 (48.1) | 47 (40.5) | 7 (41.2) | 62 (57.4) |  | | |  |  |
| ≥65 | 7,225 (76.1) | | 55 (62.5) | 7,886 (62.8) | 1,500 (68.6) | 5,688 (51.9) | 69 (59.5) | 10 (58.8) | 46 (42.6) |  | | |  |  |
| **Gender** |  | |  |  |  |  |  |  |  | <0.001 | | |  |  |
| male | 4,861 (51.2) | | 39 (44.3) | 6,707 (53.4) | 1,104 (50.5) | 5,587 (51.0) | 51 (44.0) | 10 (58.8) | 50 (46.3) |  | | |  |  |
| female | 4,629 (48.8) | | 49 (55.7) | 5,843 (46.6) | 1,081 (49.5) | 5,362 (49.0) | 65 (56.0) | 7 (41.2) | 58 (53.7) |  | | |  |  |
| **Race** |  | |  |  |  |  |  |  |  | 0.001 | | |  |  |
| Black | 742 (7.8) | | 5 (5.7) | 980 (7.8) | 235 (10.8) | 995 (9.1) | 10 (8.6) | 1 (5.9) | 13 (12.0) |  | | |  |  |
| White | 8,337 (87.9) | | 77 (87.5) | 11,036 (87.9) | 1,857 (85.0) | 9,456 (86.4) | 102 (87.9) | 16 (94.1) | 95 (88.0) |  | | |  |  |
| Asian or Pacific Islander | | 341 (3.6) | | 6 (6.8) | 452 (3.6) | 74 (3.4) | 411 (3.8) | 4 (3.4) | 0 (0.0) | 0 (0.0) |  | | |  |
| American Indian/Alaska Native | | | | 70 (0.7) | 0 (0.0) | 82 (0.7) | 19 (0.9) | 87 (0.8) | 0 (0.0) | 0 (0.0) | 0 (0.0) |  | | |
| **Married status** |  | |  |  |  |  |  |  |  | 0.005 | | |  |  |
| Married | 3,898 (41.1) | | 42 (47.7) | 6,273 (50.0) | 959 (43.9) | 5,783 (52.8) | 68 (58.6) | 7 (41.2) | 52 (48.1) |  | | |  |  |
| Divorced | 1,342 (14.1) | | 9 (10.2) | 1,816 (14.5) | 296 (13.5) | 1,579 (14.4) | 9 (7.8) | 3 (17.6) | 16 (14.8) |  | | |  |  |
| Others | 4,250 (44.8) | | 37 (42.0) | 4,461 (35.5) | 930 (42.6) | 3,587 (32.8) | 39 (33.6) | 7 (41.2) | 40 (37.0) |  | | |  |  |
| **Primary Site** |  | |  |  |  |  |  |  |  | 0.009 | | |  |  |
| Main bronchus | 939 (9.9) | | 9 (10.2) | 1,384 (11.0) | 230 (10.5) | 1,318 (12.0) | 15 (12.9) | 1 (5.9) | 20 (18.5) |  | | |  |  |
| Upper lobe | 3,726 (39.3) | | 34 (38.6) | 5,349 (42.6) | 988 (45.2) | 5,284 (48.3) | 44 (37.9) | 7 (41.2) | 48 (44.4) |  | | |  |  |
| Middle lobe | 299 (3.2) | | 4 (4.5) | 477 (3.8) | 69 (3.2) | 390 (3.6) | 5 (4.3) | 2 (11.8) | 3 (2.8) |  | | |  |  |
| Lower lobe | 1,934 (20.4) | | 24 (27.3) | 2,611 (20.8) | 447 (20.5) | 2,039 (18.6) | 32 (27.6) | 2 (11.8) | 23 (21.3) |  | | |  |  |
| Others | 2,592 (27.3) | | 17 (19.3) | 2,729 (21.7) | 451 (20.6) | 1,918 (17.5) | 20 (17.2) | 5 (29.4) | 14 (13.0) |  | | |  |  |
| **Laterality** |  | |  |  |  |  |  |  |  | 0.006 | | |  |  |
| Left | 3,714 (39.1) | | 39 (44.3) | 5,005 (39.9) | 859 (39.3) | 4,387 (40.1) | 47 (40.5) | 7 (41.2) | 42 (38.9) |  | | |  |  |
| Right | 4,754 (50.1) | | 39 (44.3) | 6,588 (52.5) | 1,158 (53.0) | 5,956 (54.4) | 61 (52.6) | 9 (52.9) | 60 (55.6) |  | | |  |  |
| Others | 1,022 (10.8) | | 10 (11.4) | 957 (7.6) | 168 (7.7) | 606 (5.5) | 8 (6.9) | 1 (5.9) | 6 (5.6) |  | | |  |  |
| **T stage** |  | |  |  |  |  |  |  |  | <0.001 | | |  |  |
| T0 | 141 (1.5) | | 0 (0.0) | 165 (1.3) | 31 (1.4) | 119 (1.1) | 0 (0.0) | 1 (5.9) | 4 (3.7) |  | | |  |  |
| T1 | 762 (8.0) | | 6 (6.8) | 1,106 (8.8) | 251 (11.5) | 1,044 (9.5) | 12 (10.3) | 2 (11.8) | 17 (15.7) |  | | |  |  |
| T2 | 1,804 (19.0) | | 22 (25.0) | 2,524 (20.1) | 468 (21.4) | 2,338 (21.4) | 27 (23.3) | 3 (17.6) | 18 (16.7) |  | | |  |  |
| T3 | 812 (8.6) | | 13 (14.8) | 1,212 (9.7) | 189 (8.6) | 1,065 (9.7) | 15 (12.9) | 4 (23.5) | 10 (9.3) |  | | |  |  |
| T4 | 4,008 (42.2) | | 30 (34.1) | 5,690 (45.3) | 941 (43.1) | 5,151 (47.0) | 49 (42.2) | 5 (29.4) | 50 (46.3) |  | | |  |  |
| TX | 1,963 (20.7) | | 17 (19.3) | 1,853 (14.8) | 305 (14.0) | 1,232 (11.3) | 13 (11.2) | 2 (11.8) | 9 (8.3) |  | | |  |  |
| **N stage** |  | |  |  |  |  |  |  |  | 0.010 | | |  |  |
| N0 | 1,450 (15.3) | | 21 (23.9) | 1,307 (10.4) | 362 (16.6) | 1,182 (10.8) | 29 (25.0) | 7 (41.2) | 21 (19.4) |  | | |  |  |
| N1 | 588 (6.2) | | 6 (6.8) | 781 (6.2) | 180 (8.2) | 728 (6.6) | 11 (9.5) | 1 (5.9) | 10 (9.3) |  | | |  |  |
| N2 | 4,646 (49.0) | | 37 (42.0) | 6,440 (51.3) | 1,044 (47.8) | 5,567 (50.8) | 52 (44.8) | 6 (35.3) | 53 (49.1) |  | | |  |  |
| N3 | 1,893 (19.9) | | 10 (11.4) | 3,392 (27.0) | 462 (21.1) | 3,028 (27.7) | 20 (17.2) | 2 (11.8) | 21 (19.4) |  | | |  |  |
| NX | 913 (9.6) | | 14 (15.9) | 630 (5.0) | 137 (6.3) | 444 (4.1) | 4 (3.4) | 1 (5.9) | 3 (2.8) |  | | |  |  |
| **Bone Metastasis** |  | |  |  |  |  |  |  |  | <0.001 | | |  |  |
| Yes | 2,742 (28.9) | | 17 (19.3) | 4,944 (39.4) | 801 (36.7) | 4,101 (37.5) | 29 (25.0) | 4 (23.5) | 29 (26.9) |  | | |  |  |
| No | 6,748 (71.1) | | 71 (80.7) | 7,606 (60.6) | 1,384 (63.3) | 6,848 (62.5) | 87 (75.0) | 13 (76.5) | 79 (73.1) |  | | |  |  |
| **Brain Metastasis** | |  |  |  |  |  |  |  |  | <0.001 | | |  |  |
| Yes | 1,410 (14.9) | | 16 (18.2) | 1,325 (10.6) | 1,295 (59.3) | 4,754 (43.4) | 12 (10.3) | 9 (52.9) | 49 (45.4) |  | | |  |  |
| No | 8,080 (85.1) | | 72 (81.8) | 11,225 (89.4) | 890 (40.7) | 6,195 (56.6) | 104 (89.7) | 8 (47.1) | 59 (54.6) |  | | |  |  |
| **Liver Metastasis** |  | |  |  |  |  |  |  |  | <0.001 | | |  |  |
| Yes | 5,365 (56.5) | | 26 (29.5) | 7,011 (55.9) | 770 (35.2) | 3,690 (33.7) | 49 (42.2) | 3 (17.6) | 26 (24.1) |  | | |  |  |
| No | 4,125 (43.5) | | 62 (70.5) | 5,539 (44.1) | 1,415 (64.8) | 7,259 (66.3) | 67 (57.8) | 14 (82.4) | 82 (75.9) |  | | |  |  |
| **Lung Metastasis** |  | |  |  |  |  |  |  |  | <0.001 | | |  |  |
| Yes | 2,110 (22.2) | | 17 (19.3) | 2,617 (20.9) | 406 (18.6) | 1,886 (17.2) | 24 (20.7) | 1 (5.9) | 15 (13.9) |  | | |  |  |
| No | 7,380 (77.8) | | 71 (80.7) | 9,933 (79.1) | 1,779 (81.4) | 9,063 (82.8) | 92 (79.3) | 16 (94.1) | 93 (86.1) |  | | |  |  |
