# Supplementary material for: Clinical features and prognostic factors of IV combined small cell lung cancer: A propensity score matching analysis
Source: PLoS One. 2024 Nov 8;19(11):e0313221. doi: 10.1371/journal.pone.0313221 (PMC11548789; doi:10.1371/journal.pone.0313221)
Supplement: S8 Table — (DOCX) [file pone.0313221.s011.docx]

S8 Table : The baseline characteristics of different treatment modalities in IV NSCLC

| **Characteristic** | **Group** | | | | | | | | |  |  |  |
| --- | --- | --- | --- | --- | --- | --- | --- | --- | --- | --- | --- | --- |
|  | **Control**  **N = 46506** | **Surgery**  **N = 1307** | **Chemotherapy**  **N = 27129** | **Radiotherapy, N = 17604** | **Chemoradiotherapy N =27792** | **Surgery**  **+chemotherapy**  **N = 1115** | **Surgery +radiotherapy**  **N = 421** | **Surgery+ chemoradiotherapy**  **N =933** | **p-value** |  |  |  |
| **Age(years)** |  |  |  |  |  |  |  |  | <0.001 |  |  |  |
| ＜65 | 12633 (27.16) | 361 (27.62) | 9693 (35.73) | 5455 (30.99) | 13265 (47.73) | 483 (43.32) | 167 (39.67) | 485 (51.98) |  |  |  |  |
| ≥65 | 33873 (72.84) | 946 (72.38) | 17436 (64.27) | 12149 (69.01) | 14527 (52.27) | 632 (56.68) | 254 (60.33) | 448 (48.02) |  |  |  |  |
| **Gender** |  |  |  |  |  |  |  |  | <0.001 |  |  |  |
| male | 25247 (54.29) | 662 (50.65) | 14505 (53.47) | 9950 (56.52) | 14970 (53.86) | 556 (49.87) | 223 (52.97) | 491 (52.63) |  |  |  |  |
| female | 21259 (45.71) | 645 (49.35) | 12624 (46.53) | 7654 (43.48) | 12822 (46.14) | 559 (50.13) | 198 (47.03) | 442 (47.37) |  |  |  |  |
| **Race** |  |  |  |  |  |  |  |  | 0.009 |  |  |  |
| Black | 5569 (11.97) | 134 (10.25) | 3219 (11.87) | 2370 (13.46) | 3365 (12.11) | 134 (12.02) | 44 (10.45) | 95 (10.18) |  |  |  |  |
| White | 36200 (77.84) | 1086 (83.09) | 20588 (75.89) | 14073 (79.94) | 21606 (77.74) | 873 (78.30) | 363 (86.22) | 786 (84.24) |  |  |  |  |
| Asian or Pacific Islander | | | 4415 (9.49) | 78 (5.97) | 3200 (11.80) | 1058 (6.01) | 2693 (9.69) | 105 (9.42) | 11 (2.61) | 50 (5.36) |  |  |
| American Indian/Alaska Native | | | | 322 (0.69) | 9 (0.69) | 122 (0.45) | 103 (0.59) | 128 (0.46) | 3 (0.27) | 3 (0.71) | 2 (0.21) |  |
| **Married status** |  |  |  |  |  |  |  |  | <0.001 |  |  |  |
| Married | 20854 (44.84) | 687 (52.56) | 15119 (55.73) | 8130 (46.18) | 15789 (56.81) | 630 (56.50) | 226 (53.68) | 542 (58.09) |  |  |  |  |
| Divorced | 5818 (12.51) | 143 (10.94) | 2837 (10.46) | 2277 (12.93) | 3370 (12.13) | 149 (13.36) | 56 (13.30) | 104 (11.15) |  |  |  |  |
| Others | 19834 (42.65) | 477 (36.50) | 9173 (33.81) | 7197 (40.88) | 8633 (31.06) | 336 (30.13) | 139 (33.02) | 287 (30.76) |  |  |  |  |
| **Primary Site** |  |  |  |  |  |  |  |  | 0.009 |  |  |  |
| Main bronchus | 1868 (4.02) | 41 (3.14) | 897 (3.31) | 872 (4.95) | 1371 (4.93) | 23 (2.06) | 27 (6.41) | 59 (6.32) |  |  |  |  |
| Upper lobe | 20939 (45.02) | 578 (44.22) | 12631 (46.56) | 9036 (51.33) | 15015 (54.03) | 482 (43.23) | 207 (49.17) | 502 (53.80) |  |  |  |  |
| Middle lobe | 1761 (3.79) | 76 (5.81) | 1134 (4.18) | 696 (3.95) | 1145 (4.12) | 64 (5.74) | 20 (4.75) | 44 (4.72) |  |  |  |  |
| Lower lobe | 11493 (24.71) | 373 (28.54) | 7360 (27.13) | 4582 (26.03) | 6973 (25.09) | 333 (29.87) | 127 (30.17) | 226 (24.22) |  |  |  |  |
| Others | 10445 (22.46) | 239 (18.29) | 5107 (18.82) | 2418 (13.74) | 3288 (11.83) | 213 (19.10) | 40 (9.50) | 102 (10.93) |  |  |  |  |
| **Laterality** |  |  |  |  |  |  |  |  | <0.001 |  |  |  |
| Left | 17409 (37.43) | 503 (38.49) | 10585 (39.02) | 7010 (39.82) | 10934 (39.34) | 419 (37.58) | 166 (39.43) | 380 (40.73) |  |  |  |  |
| Right | 25035 (53.83) | 764 (58.45) | 14733 (54.31) | 9619 (54.64) | 15716 (56.55) | 658 (59.01) | 243 (57.72) | 528 (56.59) |  |  |  |  |
| Others | 4062 (8.73) | 40 (3.06) | 1811 (6.68) | 975 (5.54) | 1142 (4.11) | 38 (3.41) | 12 (2.85) | 25 (2.68) |  |  |  |  |
| **T stage** |  |  |  |  |  |  |  |  | 0.006 |  |  |  |
| T0 | 505 (1.09) | 1 (0.08) | 238 (0.88) | 153 (0.87) | 197 (0.71) | 1 (0.09) | 0 (0.00) | 6 (0.64) |  |  |  |  |
| T1 | 4528 (9.74) | 272 (20.81) | 2918 (10.76) | 2245 (12.75) | 3422 (12.31) | 162 (14.53) | 73 (17.34) | 151 (16.18) |  |  |  |  |
| T2 | 10032 (21.57) | 390 (29.84) | 6183 (22.79) | 4227 (24.01) | 7305 (26.28) | 319 (28.61) | 137 (32.54) | 306 (32.80) |  |  |  |  |
| T3 | 4177 (8.98) | 137 (10.48) | 2896 (10.67) | 2205 (12.53) | 3474 (12.50) | 159 (14.26) | 56 (13.30) | 142 (15.22) |  |  |  |  |
| T4 | 19913 (42.82) | 426 (32.59) | 11473 (42.29) | 6902 (39.21) | 10962 (39.44) | 421 (37.76) | 135 (32.07) | 287 (30.76) |  |  |  |  |
| TX | 7351 (15.81) | 81 (6.20) | 3421 (12.61) | 1872 (10.63) | 2432 (8.75) | 53 (4.75) | 20 (4.75) | 41 (4.39) |  |  |  |  |
| **N stage** |  |  |  |  |  |  |  |  | <0.001 |  |  |  |
| N0 | 12540 (26.96) | 781 (59.76) | 5771 (21.27) | 4691 (26.65) | 5462 (19.65) | 469 (42.06) | 197 (46.79) | 337 (36.12) |  |  |  |  |
| N1 | 3272 (7.04) | 130 (9.95) | 1969 (7.26) | 1436 (8.16) | 2315 (8.33) | 183 (16.41) | 42 (9.98) | 128 (13.72) |  |  |  |  |
| N2 | 17980 (38.66) | 248 (18.97) | 10747 (39.61) | 7194 (40.87) | 12200 (43.90) | 312 (27.98) | 128 (30.40) | 360 (38.59) |  |  |  |  |
| N3 | 8529 (18.34) | 82 (6.27) | 7042 (25.96) | 3274 (18.60) | 6718 (24.17) | 101 (9.06) | 36 (8.55) | 88 (9.43) |  |  |  |  |
| NX | 4185 (9.00) | 66 (5.05) | 1600 (5.90) | 1009 (5.73) | 1097 (3.95) | 50 (4.48) | 18 (4.28) | 20 (2.14) |  |  |  |  |
| **Bone Metastasis** | |  |  |  |  |  |  |  |  | <0.001 |  |  |
| Yes | 16797 (36.12) | 171 (13.08) | 9839 (36.27) | 8690 (49.36) | 13420 (48.29) | 171 (15.34) | 110 (26.13) | 233 (24.97) |  |  |  |  |
| No | 29709 (63.88) | 1136 (86.92) | 17290 (63.73) | 8914 (50.64) | 14372 (51.71) | 944 (84.66) | 311 (73.87) | 700 (75.03) |  |  |  |  |
| **Brain Metastasis** | |  |  |  |  |  |  |  |  | <0.001 |  |  |
| Yes | 8659 (18.62) | 130 (9.95) | 2380 (8.77) | 7865 (44.68) | 12475 (44.89) | 78 (7.00) | 190 (45.13) | 378 (40.51) |  |  |  |  |
| No | 37847 (81.38) | 1177 (90.05) | 24749 (91.23) | 9739 (55.32) | 15317 (55.11) | 1037 (93.00) | 231 (54.87) | 555 (59.49) |  |  |  |  |
| **Liver Metastasis** | |  |  |  |  |  |  |  |  | <0.001 |  |  |
| Yes | 8843 (19.01) | 61 (4.67) | 5007 (18.46) | 2875 (16.33) | 4124 (14.84) | 78 (7.00) | 40 (9.50) | 49 (5.25) |  |  |  |  |
| No | 37663 (80.99) | 1246 (95.33) | 22122 (81.54) | 14729 (83.67) | 23668 (85.16) | 1037 (93.00) | 381 (90.50) | 884 (94.75) |  |  |  |  |
| **Lung Metastasis** | |  |  |  |  |  |  |  |  | <0.001 |  |  |
| Yes | 15480 (33.29) | 344 (26.32) | 10201 (37.60) | 4761 (27.04) | 7206 (25.93) | 309 (27.71) | 74 (17.58) | 193 (20.69) |  |  |  |  |
| No | 31026 (66.71) | 963 (73.68) | 16928 (62.40) | 12843 (72.96) | 20586 (74.07) | 806 (72.29) | 347 (82.42) | 740 (79.31) |  |  |  |  |
