# Supplementary material for: Clinical features and prognostic factors of IV combined small cell lung cancer: A propensity score matching analysis
Source: PLoS One. 2024 Nov 8;19(11):e0313221. doi: 10.1371/journal.pone.0313221 (PMC11548789; doi:10.1371/journal.pone.0313221)
Supplement: S9 Table — * OS adjusted for Age(years), Race, N stage, Bone Metastasis, and Liver Metastasis, *CSS adjusted for Age(years), Race, T stage, N stage, Bone Metastasis, and Liver Metastasis. (DOCX) [file pone.0313221.s012.docx]

S9 Table : Cox regression for efficacy analysis of IV CSCLC

| **therapy methods** | **OS** | | | **CSS** | |
| --- | --- | --- | --- | --- | --- |
|  | **HR(95CI)** | **P value** | **HR(95CI)** | | **P Value** |
| **Unadjusted** |  |  |  | |  |
| **Control** | — |  | — | |  |
| **Surgery** | 0.27(0.12, 0.58) | <0.001 | 0.32(0.15, 0.70) | | 0.004 |
| **Chemotherapy** | 0.27(0.21, 0.35) | <0.001 | 0.31(0.24, 0.41) | | <0.001 |
| **Radiotherapy** | 0.52(0.37, 0.73) | <0.001 | 0.57(0.40, 0.82) | | 0.002 |
| **Chemoradiotherapy** | 0.24(0.18, 0.31) | <0.001 | 0.27(0.20, 0.35) | | <0.001 |
| **Surgery+ chemotherapy** | 0.19(0.09, 0.40) | <0.001 | 0.18(0.08, 0.40) | | <0.001 |
| **Surgery + radiotherapy** | NA | NA | NA | | NA |
| **Surgery+ chemoradiotherapy** | 0.13(0.06, 0.29) | <0.001 | 0.16(0.08, 0.36) | | <0.001 |
| **Adjusted*** |  |  |  | |  |
| **Control** | — |  | — | |  |
| **Surgery** | 0.24(0.11, 0.53) | <0.001 | 0.29(0.13, 0.63) | | 0.002 |
| **Chemotherapy** | 0.23(0.17, 0.30) | <0.001 | 0.26(0.19, 0.35) | | <0.001 |
| **Radiotherapy** | 0.53(0.38, 0.76) | <0.001 | 0.55(0.38, 0.81) | | 0.002 |
| **Chemoradiotherapy** | 0.21(0.16, 0.27) | <0.001 | 0.22(0.17, 0.30) | | <0.001 |
| **Surgery+ chemotherapy** | 0.18(0.09, 0.37) | <0.001 | 0.15(0.07, 0.36) | | <0.001 |
| **Surgery + radiotherapy** | NA | NA | NA | | NA |
| **Surgery+ chemoradiotherapy** | 0.16(0.07, 0.36) | <0.001 | 0.21(0.09, 0.48) | | <0.001 |
| * OS adjusted for Age(years), Race, N stage, Bone Metastasis, and Liver Metastasis  *CSS adjusted for Age(years), Race, T stage, N stage, Bone Metastasis, and Liver Metastasis | | | | | |
