# Supplementary material for: Clinical features and prognostic factors of IV combined small cell lung cancer: A propensity score matching analysis
Source: PLoS One. 2024 Nov 8;19(11):e0313221. doi: 10.1371/journal.pone.0313221 (PMC11548789; doi:10.1371/journal.pone.0313221)
Supplement: S10 Table — * OS and CSS adjusted for for Age(years), Gender, Race, Married status, Primary Site, T stage, N stage, Bone Metastasis, Brain Metastasis, Liver Metastasis, and Lung Metastasis. (DOCX) [file pone.0313221.s013.docx]

S10 Table : Cox regression for efficacy analysis of IV SCLC

| **therapy methods** | **OS** | | | **CSS** | |
| --- | --- | --- | --- | --- | --- |
|  | **HR(95CI)** | **P value** | **HR(95CI)** | | **P Value** |
| **Unadjusted** |  |  |  | |  |
| **Control** | — |  | — | |  |
| **Surgery** | 0.39（0.31, 0.48） | <0.001 | 0.4（0.32, 0.50） | | <0.001 |
| **Chemotherapy** | 0.26（0.25, 0.27） | <0.001 | 0.27（0.26, 0.28） | | <0.001 |
| **Radiotherapy** | 0.61（0.58, 0.64） | <0.001 | 0.64（0.61, 0.68） | | <0.001 |
| **Chemoradiotherapy** | 0.18（0.17, 0.18） | <0.001 | 0.19（0.18, 0.19） | | <0.001 |
| **Surgery+ chemotherapy** | 0.16（0.13, 0.20） | <0.001 | 0.17（0.14, 0.21） | | <0.001 |
| **Surgery + radiotherapy** | 0.75（0.46, 1.20） | 0.002 | 0.8（0.49, 1.31） | | 0.005 |
| **Surgery+ chemoradiotherapy** | 0.13（0.11, 0.16） | <0.001 | 0.14（0.11, 0.17） | | <0.001 |
| **Adjusted*** |  |  |  | |  |
| **Control** | — |  | — | |  |
| **Surgery** | 0.47（0.37, 0.58） | <0.001 | 0.47（0.37, 0.59） | | <0.001 |
| **Chemotherapy** | 0.25（0.24, 0.25） | <0.001 | 0.25（0.24, 0.26） | | <0.001 |
| **Radiotherapy** | 0.6（0.57, 0.63） | <0.001 | 0.63（0.60, 0.66） | | <0.001 |
| **Chemoradiotherapy** | 0.17（0.17, 0.18） | <0.001 | 0.17（0.17, 0.18） | | <0.001 |
| **Surgery+ chemotherapy** | 0.18（0.15, 0.22） | <0.001 | 0.18（0.15, 0.22） | | <0.001 |
| **Surgery + radiotherapy** | 0.76（0.48, 1.23） | 0.003 | 0.8（0.49, 1.31） | | 0.007 |
| **Surgery+ chemoradiotherapy** | 0.14（0.12, 0.17） | <0.001 | 0.14（0.11, 0.17） | | <0.001 |
| * OS and CSS adjusted for for Age(years), Gender, Race, Married status, Primary Site, T stage, N stage, Bone Metastasis, Brain Metastasis, Liver Metastasis, and Lung Metastasis | | | | | |

S11
