# Supplementary material for: Clinical features and prognostic factors of IV combined small cell lung cancer: A propensity score matching analysis
Source: PLoS One. 2024 Nov 8;19(11):e0313221. doi: 10.1371/journal.pone.0313221 (PMC11548789; doi:10.1371/journal.pone.0313221)
Supplement: S11 Table — * OS and CSS adjusted for Age years, Gender, Race, Married status, Primary Site, T stage, N stage, Bone Metastasis, Brain Metastasis, Liver Metastasis, and Lung Metastasis. (DOCX) [file pone.0313221.s014.docx]

S11 Table : Cox regression for efficacy analysis of IV NSCLC

| **therapy methods** | **OS** | | | **CSS** | |
| --- | --- | --- | --- | --- | --- |
|  | **HR(95CI)** | **P value** | **HR(95CI)** | | **P Value** |
| **Unadjusted** |  |  |  | |  |
| **Control** | — |  | — | |  |
| **Surgery** | 0.36（0.33, 0.38） | <0.001 | 0.33(0.31, 0.35) | | <0.001 |
| **Chemotherapy** | 0.44（0.43, 0.45） | <0.001 | 0.45 (0.44, 0.46) | | <0.001 |
| **Radiotherapy** | 0.95（0.93, 0.97） | <0.001 | 0.97 (0.95, 0.99) | | 0.005 |
| **Chemoradiotherapy** | 0.47（0.46, 0.48） | <0.001 | 0.49 (0.48, 0.50) | | <0.001 |
| **Surgery+ chemotherapy** | 0.24（0.23, 0.26） | <0.001 | 0.24 (0.22, 0.26) | | <0.001 |
| **Surgery + radiotherapy** | 0.48（0.43, 0.54） | <0.001 | 0.48 (0.43, 0.54) | | <0.001 |
| **Surgery+ chemoradiotherapy** | 0.29（0.27, 0.31） | <0.001 | 0.30 (0.28, 0.32) | | <0.001 |
| **Adjusted*** |  |  |  | |  |
| **Control** | — |  | —— | |  |
| **Surgery** | 0.45（0.42, 0.48） | <0.001 | 0.43 (0.40, 0.46) | | <0.001 |
| **Chemotherapy** | 0.43（0.43, 0.44） | <0.001 | 0.44 (0.43, 0.45) | | <0.001 |
| **Radiotherapy** | 0.92（0.90, 0.93） | <0.001 | 0.93 (0.91, 0.95) | | <0.001 |
| **Chemoradiotherapy** | 0.45（0.44, 0.46） | <0.001 | 0.46 (0.45, 0.47) | | <0.001 |
| **Surgery+ chemotherapy** | 0.29（0.27, 0.31） | <0.001 | 0.28 (0.26, 0.31) | | <0.001 |
| **Surgery + radiotherapy** | 0.54（0.48, 0.60） | <0.001 | 0.54 (0.48, 0.61) | | <0.001 |
| **Surgery+ chemoradiotherapy** | 0.32（0.30, 0.35） | <0.001 | 0.33 (0.31, 0.36) | | <0.001 |
| * OS and CSS adjusted for Age years, Gender, Race, Married status, Primary Site, T stage, N stage, Bone Metastasis, Brain Metastasis, Liver Metastasis, and Lung Metastasis | | | | | |
