# Supplementary material for: Clinical features and prognostic factors of IV combined small cell lung cancer: A propensity score matching analysis
Source: PLoS One. 2024 Nov 8;19(11):e0313221. doi: 10.1371/journal.pone.0313221 (PMC11548789; doi:10.1371/journal.pone.0313221)
Supplement: Attachment — Submitted filename: response to reviewers.docx [file pone.0313221.s015.docx]

**Dear esteemed Editor and Reviewers,**

I am writing to express my sincere gratitude for the invaluable time and effort you have dedicated to reviewing my manuscript “*Clinical features and prognostic factors of IV combined small cell lung cancer: A Propensity Score Matching Analysis"* (manuscript number: PONE-D-24-30493)”. Your insightful comments and suggestions have been instrumental in enhancing the quality and clarity of my work.

I have thoroughly revised the manuscript in accordance with your guidelines and recommendations. In addition to addressing each of your specific suggestions, I have also conducted a careful review for grammatical errors, further refining the text for improved readability. To facilitate your review of the changes, I have highlighted the corrections for grammatical errors in yellow and marked the revisions that directly respond to the reviewers' comments in red. Our responses are outlined below, and the corrections made to the manuscript are explained.

**Editor** **Requirements:**

1. **Please ensure that your manuscript meets PLOS ONE's style requirements, including those for file naming.**

**Reply:** Thanks to your valuable suggestion, I have reformatted the manuscript to conform precisely to PLOS ONE's specifications, including adjustments to font size, margins, line spacing, reference format, etc. The file naming convention has been strictly followed for both the manuscript and any accompanying materials.

2. **Please note that funding information should not appear in any section or other areas of your manuscript. We will only publish funding information present in the Funding Statement section of the online submission form. Please remove any funding-related text from the manuscript.**

**Reply:** Thank you for your advice. I have deleted the funding information part in the manuscript.

3. **We note that the grant information you provided in the ‘Funding Information’and ‘Financial Disclosure’ sections do not match. When you resubmit, please ensure that you provide the correct grant numbers for the awards you received for your study in the ‘Funding Information’ section.**

**Reply:** Thank you for your careful review. We apologize for our previous oversight. we modified Funding Information and Financial Disclosure.

4. **Please provide a complete Data Availability Statement in the submission form, ensuring you include all necessary access information or a reason for why you are unable to make your data freely accessible. If your research concerns only data provided within your submission, please write "All data are in the manuscript and/or supporting information files" as your Data Availability Statement.**

**Reply:** Thanks, I have provided a complete Data Availability Statement in the submission form.

5. **PLOS requires an ORCID iD for the corresponding author in Editorial Manager on papers submitted after December 6th, 2016. Please ensure that you have an ORCID iD and that it is validated in Editorial Manager. To do this, go to ‘Update my Information’ (in the upper left-hand corner of the main menu), and click on the Fetch/Validate link next to the ORCID field. This will take you to the ORCID site and allow you to create a new iD or authenticate a pre-existing iD in Editorial Manager.**

**Reply:** Thanks, I have included the ORCID ID of the corresponding author to enhance discoverability and credit attribution.

6. **Please amend the manuscript submission data (via Edit Submission) to include author Zhouhua Li , Xiaotian Huang and Jinbo Li.**

**Reply:** Thanks, I have reviewed and updated authors information to ensure its accuracy and completeness, adhering to PLOS ONE's requirements.

7. **We note you have included a table to which you do not refer in the text of your manuscript. Please ensure that you refer to Table 5 in your text; if accepted, production will need this reference to link the reader to the Table.**

**Reply:** Thank you for your reminder. After substantial revisions to the full text, I have carefully checked it to ensure that such an issue will not arise. All figures and tables have been properly cited.

**Reviewer's comments:**

**Reviewer #1**

1. **Instead of using performance status or comorbidities, the authors used initial treatment and metastatic status as variables for PSM. These variables could be confounding, as the initial treatment may not be independent of metastatic status.**

**Reply:** Thank you for your suggestions. In this study, I have incorporated the performance status (including age, gender, race, marital status, laterality, and primary site) and stage (T stage, N stage) as per your request. However, after meticulous contemplation and thorough literature review, I discovered that both the metastatic states (including brain, bone, liver, and lung metastasis) and the initial treatments (surgery, radiotherapy, chemotherapy), along with the aforementioned balancing variables[1-12] can significantly impact the survival outcomes of lung cancer patients. Our objective in PSM is to ensure the comparability of survival outcomes across the three patient groups. Given that these factors exert a pivotal influence on patients' survival, failing to include them in the analysis could potentially introduce substantial bias into subsequent statistical comparisons. Indeed, my subsequent univariate and multivariate Cox regression analyses on the primary survival outcomes further validated that the metastatic status and initial treatments are independent risk and protective factors, respectively, for the three groups. Furthermore, upon reviewing the literature, I found that previous studies in this domain consistently incorporated the metastatic status and initial treatment as influential variables within their PSM analyses[13-17]. Consequently, based on these considerations，I have decided to include these two types of variables in my own analysis as well. All the references mentioned above are attached at the end of this paper for further verification and reference.

2. **Regarding the Kaplan-Meier analysis, the results of "Before PSM" and "After PSM" are mixed within the chapter, making it difficult for readers to understand. Please separate the sections for "Before PSM" and "After PSM" in the chapter for both patient characteristics and KM analysis.**

**Reply:** Thank you sincerely for your insightful suggestions. I have segregated the results into distinct sections, clearly differentiating between those obtained before and after PSM. Furthermore, I have meticulously redrawn the survival curves and segregated them into two distinct figures (Figure 4 and Figure 5) to clearly illustrate the before PSM and after PSM survival outcomes among three groups. To facilitate easy identification, the revised content has been conspicuously highlighted in red font throughout the text.

3. **Additionally, the authors are conducting two analyses: CSCLC vs. SCLC and CSCLC vs. NSCLC. Since the CSCLC populations in each analysis are different, the authors should not present the results of three cohorts in parallel within the same sections, as this could confuse readers and lead to misleading conclusions.**

**Reply:** Thank you for your reminder. To mitigate bias and ensure the rigorousness of our research, I have revised the study design to adopt a 1:4 PSM, significantly expanding the sample sizes for both IV NSCLC and IV SCLC（Tables 2,3 and Fig 2）. This adjustment included all CSCLC samples (n=493) and the problem of different CSCLC populations in each analysis was solved.

During the analysis of risk factors, because the population of CSCLC is the same every time, isolating CSCLC against SCLC and NSCLC individually would result in redundant occurrences of the same CSCLC outcomes, unnecessarily complicating the process. Consequently, so the three groups are presented within a single table for clarity (Table 5). Nevertheless, in the subsequent result interpretation, apart from some influencing factors shared by the three groups, we also conduct distinct analyses comparing CSCLC versus SCLC and CSCLC versus NSCLC, respectively, the revised content has been conspicuously highlighted in red font throughout the text and table.

4. **Patients who received radiotherapy as the initial treatment in stage IV were symptomatic and received palliative radiotherapy, typically associated with poor prognosis in clinical practice. In contrast, those who underwent surgery were likely patients with relatively localized metastatic disease or in good PS. This could introduce significant bias in comparisons, so PSM is usually employed for such comparisons. However, the main focused PSM of this study is on CSCLC vs. SCLC and CSCLC vs. NSCLC, and another PSM is required to demonstrate the authors’ hypothesis. The sections on "** **the prognosis of each treatment modality in IV CSCLC patients" and "the evaluation of different treatment modalities" would be aside from the main objectives in this study, and the statistical method is not appropriate, even though it is a subsequent analysis. The authors should reconsider the main objectives and results of this study in conjunction with the statistical methods used.**

**Reply:** Thank you sincerely for your insightful suggestions. I have adjusted statistical method for the two sections, the corresponding statistical methods, results and discussion sections have been revised too. The revised content has been conspicuously highlighted in red font throughout the text and table.

For the section on *“the prognosis of each treatment modality in IV CSCLC patients”*, PSM was applied to each treatment modality in IV CSCLC patients in this study per your request. For surgery vs. no surgery groups, a 1:4 matching ratio with a 0.02 caliper value was adopted using the “nearest” method (S1A Fig, S3 Table). Similarly, 1:1 ratio and 0.02 caliper values were applied to radiotherapy vs. no radiotherapy groups (S1B Fig, S4 Table) and chemotherapy vs. no chemotherapy groups (S1C Fig, S5 Table), also utilizing the "nearest" method. All covariates were subsequently well balanced (p≥0.05 and SMD＜0.1). Matching variables encompassed age, gender, race, marital status, T stage, N stage, laterality, primary site, brain metastasis, bone metastasis, liver metastasis, lung metastasis, along with other therapies received (surgery /radiotherapy/ chemotherapy). In addition, we also added the survival curve after PSM (Fig 10)

For the section on *“Evaluation of different treatment modalities of IV CSCLC, IV SCLC and IV NSCLC”*, For comparison among baseline characteristics of different treatment modalities, we used the Fisher exact test for expected frequencies of <5, otherwise, we used the Chi-squared test. Kaplan-Meier method and log-rank test were used to compare the prognosis of different treatment modalities, we revised the table about mOS and mCSS of different therapy methods in three groups, the median survival (95% CI) and log-rank P value compared with control group were included in the tables 6-8. In addition, Adjusted analyses for the primary survival outcome of different treatment modalities of IV CSCLC, IV SCLC and IV NSCLC were performed using Cox regression models to estimated HRs with corresponding two sides 95CIs, considering potential unbalanced confounders that may have influenced the outcomes (S9-S11 Tables). The survival curve after adjusted was shown in S3 Fig. The covariates included in the adjustment were confounding factors that were found by cox regression analysis to be likely to influence prognosis. The adjusted covariates in each group were described in each table legend.

**Reviewer #2**

1. **In table 1 before PSM, there are so many P value <0.05, but in table 2 after PSM, there are less sample size, which will cause bias, please explain it.**

**Reply:** Thank you sincerely for your insightful suggestions. To mitigate potential biases and ensure the rigorousness of our research. I have revised the study design to change 1:1 PSM to 1:4 PSM, significantly expanding the sample sizes for both IV NSCLC and IV SCLC. All covariates were subsequently well balanced both in the 1:4 matched cohort of IV CSCLC (n=493) vs IV SCLC (n=1954), and the 1:4 matched cohort of IV CSCLC (n=493) vs IV NSCLC (n=1968). (p≥0.05 and SMD＜0.1, Table2,3, Fig 2). We also modified the subsequent research results accordingly; the revised content has been conspicuously highlighted in red font throughout the text and table.

2. **all figs are low quality, please improve the absolution.**

**Reply:** Thank you for your suggestion. I have redrawn all the figures and ensured that they meet the journal requirements of PLOS ONE by maintaining a resolution of above 300 ppi. All figures have been uploaded in TIF format.

3. **the conclusion should summarize more concisely and logically. you indicate "we found that the prognosis of IV CSCLC patients was significantly worse than that of IV NSCLC patients, but better than IV SCLC patients." please provide evidence.**

**Reply:** Thanks, I have revised the section of conclusion and corrected the imprecise statement as your request with highlighted in red. Importantly, I have revised “we found that the prognosis of IV CSCLC patients was significantly worse than that of IV NSCLC patients, but better than IV SCLC patients” to “the prognosis for patients with IV CSCLC was notably inferior to that of patients with IV NSCLC, but was similar to IV SCLC”. This conclusion is elaborated upon in detail in section “KM analysis for IV CSCLC, IV SCLC and IV NSCLC before PSM” and section “KM analysis for IV CSCLC, IV SCLC and IV NSCLC after PSM”, with modifications highlighted in red.

4. **the format of study should revise according scientific format. Please check it.**

**Reply:** Thanks to your valuable suggestion, I have reformatted the manuscript to conform precisely to PLOS ONE's specifications, including adjustments to font size, margins, line spacing, reference format, etc. Tables and figures were also redrawn as required. In addition, I have also conducted a careful review for grammatical errors, which highlighted in yellow.

5. **Table 5: The mOS and mCSS of different therapy methods in IV CSCLC, SCLC and IV SCLC, there is no statistic analysis to show in table, you should compare to analysize to achieve the conclusion.**

**Reply:** Thanks to your valuable suggestion. In our study, Kaplan-Meier method and log-rank test were used to compare the prognosis of different treatment modalities, we revised the table about mOS and mCSS of different therapy methods in three groups per your request, the median survival (95% CI) and log-rank P value compared with control group were included in the tables 6-8. In addition, adjusted analyses for the primary survival outcome of different treatment modalities of IV CSCLC, IV SCLC and IV NSCLC were performed using Cox regression models to estimated HRs with corresponding two sides 95CIs, considering potential unbalanced confounders that may have influenced the outcomes (S9-S11 Tables). The revised content has been conspicuously highlighted in red font throughout the text and table.

Thanks again to the Editor and Reviewer for their valuable comments. We now resubmit the revised manuscript and hope that all corrections are satisfactory. We look forward to hearing from you regarding our submission. We would be glad to respond to any further questions and comments that you may have.

Best wishes!

Yours Sincerely

Xiaoqun Ye

**Reference**

1.	Hemminki K, Riihimäki M, Sundquist K, Hemminki A. Site-specific survival rates for cancer of unknown primary according to location of metastases. International journal of cancer. 2013;133(1):182-9. Epub 2012/12/13. doi: 10.1002/ijc.27988. PubMed PMID: 23233409.

2.	Jakobsen E, Rasmussen TR, Green A. Mortality and survival of lung cancer in Denmark: Results from the Danish Lung Cancer Group 2000-2012. Acta oncologica (Stockholm, Sweden). 2016;55 Suppl 2:2-9. Epub 2016/04/09. doi: 10.3109/0284186x.2016.1150608. PubMed PMID: 27056247.

3.	Kehl KL, Lathan CS, Johnson BE, Schrag D. Race, Poverty, and Initial Implementation of Precision Medicine for Lung Cancer. Journal of the National Cancer Institute. 2019;111(4):431-4. Epub 2018/12/24. doi: 10.1093/jnci/djy202. PubMed PMID: 30576459; PubMed Central PMCID: PMCPMC6449167.

4.	Meza R, Meernik C, Jeon J, Cote ML. Lung cancer incidence trends by gender, race and histology in the United States, 1973-2010. PloS one. 2015;10(3):e0121323. Epub 2015/03/31. doi: 10.1371/journal.pone.0121323. PubMed PMID: 25822850; PubMed Central PMCID: PMCPMC4379166.

5.	Riihimäki M, Hemminki A, Fallah M, Thomsen H, Sundquist K, Sundquist J, et al. Metastatic sites and survival in lung cancer. Lung cancer (Amsterdam, Netherlands). 2014;86(1):78-84. Epub 2014/08/19. doi: 10.1016/j.lungcan.2014.07.020. PubMed PMID: 25130083.

6.	Shugarman LR, Mack K, Sorbero ME, Tian H, Jain AK, Ashwood JS, et al. Race and sex differences in the receipt of timely and appropriate lung cancer treatment. Medical care. 2009;47(7):774-81. Epub 2009/06/19. doi: 10.1097/MLR.0b013e3181a393fe. PubMed PMID: 19536007.

7.	Torre LA, Siegel RL, Jemal A. Lung Cancer Statistics. Advances in experimental medicine and biology. 2016;893:1-19. Epub 2015/12/17. doi: 10.1007/978-3-319-24223-1_1. PubMed PMID: 26667336.

8.	Wang H, Shui L, Chen R, Chen Y, Guo J, Chen Y. Occurrence and prognosis of lung cancer metastasis to major organs: a population-based study. European journal of cancer prevention : the official journal of the European Cancer Prevention Organisation (ECP). 2023;32(3):246-53. Epub 2023/03/11. doi: 10.1097/cej.0000000000000785. PubMed PMID: 36896839.

9.	Nur U, Quaresma M, De Stavola B, Peake M, Rachet B. Inequalities in non-small cell lung cancer treatment and mortality. Journal of epidemiology and community health. 2015;69(10):985-92. Epub 2015/06/07. doi: 10.1136/jech-2014-205309. PubMed PMID: 26047831; PubMed Central PMCID: PMCPMC4602267.

10.	Bryan DS, Donington JS. The Role of Surgery in Management of Locally Advanced Non-Small Cell Lung Cancer. Current treatment options in oncology. 2019;20(4):27. Epub 2019/03/16. doi: 10.1007/s11864-019-0624-7. PubMed PMID: 30874964.

11.	Johnson ML, Patel JD. Chemotherapy and targeted therapeutics as maintenance of response in advanced non-small cell lung cancer. Seminars in oncology. 2014;41(1):93-100. Epub 2014/02/26. doi: 10.1053/j.seminoncol.2013.12.007. PubMed PMID: 24565583.

12.	Chan C, Lang S, Rowbottom C, Guckenberger M, Faivre-Finn C. Intensity-modulated radiotherapy for lung cancer: current status and future developments. Journal of thoracic oncology : official publication of the International Association for the Study of Lung Cancer. 2014;9(11):1598-608. Epub 2014/12/02. doi: 10.1097/jto.0000000000000346. PubMed PMID: 25436795.

13.	Zou L, Guo T, Ye L, Zhou Y, Chu L, Chu X, et al. Outcomes for Surgery in Stage IA Large Cell Lung Neuroendocrine Compared With Other Types of Non-Small Cell Lung Cancer: A Propensity Score Matching Study Based on the Surveillance, Epidemiology, and End Results (SEER) Database. Frontiers in oncology. 2020;10:572462. Epub 2020/12/17. doi: 10.3389/fonc.2020.572462. PubMed PMID: 33324549; PubMed Central PMCID: PMCPMC7727448.

14.	Huang L, Feng Y, Xie T, Zhu H, Tang L, Shi Y. Incidence, survival comparison, and novel prognostic evaluation approaches for stage iii-iv pulmonary large cell neuroendocrine carcinoma and small cell lung cancer. BMC cancer. 2023;23(1):312. Epub 2023/04/06. doi: 10.1186/s12885-023-10797-3. PubMed PMID: 37020179; PubMed Central PMCID: PMCPMC10077650.

15.	Yang W, Wang W, Li Z, Wu J, Xu X, Chen C, et al. Differences between Advanced Large Cell Neuroendocrine Carcinoma and Advanced Small Cell Lung Cancer: A Propensity Score Matching Analysis. Journal of Cancer. 2023;14(9):1541-52. Epub 2023/06/16. doi: 10.7150/jca.84600. PubMed PMID: 37325062; PubMed Central PMCID: PMCPMC10266246.

16.	Lv SY, Lin MJ, Yang ZQ, Xu CN, Wu ZM. Survival Analysis and Prediction Model of ASCP Based on SEER Database. Frontiers in oncology. 2022;12:909257. Epub 2022/07/12. doi: 10.3389/fonc.2022.909257. PubMed PMID: 35814413; PubMed Central PMCID: PMCPMC9263703.

17.	Chen M, Yang Q, Xu Z, Luo B, Li F, Yu Y, et al. Survival Analysis and Prediction Model for Pulmonary Sarcomatoid Carcinoma Based on SEER Database. Frontiers in oncology. 2021;11:630885. Epub 2021/06/18. doi: 10.3389/fonc.2021.630885. PubMed PMID: 34136380; PubMed Central PMCID: PMCPMC8201495.
